# WTAP Mediated m6A Modification Stabilizes PDIA3P1 and Promotes Tumor Progression Driven by Histone Lactylation in Esophageal Squamous Cell Carcinoma

**DOI:** 10.1002/advs.202506529

**Published:** 2025-06-05

**Authors:** Tao Huang, Qi You, Jiawei Liu, Xuguang Shen, Dengjun Huang, Xinlu Tao, Zhijie He, Chengwei Wu, Xinran Xi, Shouqiang Yu, Feng Liu, Zhihao Wu, Wenjun Mao, Shaojin Zhu

**Affiliations:** ^1^ Department of Thoracic Surgery The First Affiliated Hospital of Wannan Medical College (Yijishan Hospital of Wannan Medical College) Wuhu 241001 China; ^2^ Department of Thoracic Surgery The Affiliated Wuxi People's Hospital of Nanjing Medical University Wuxi People's Hospital Wuxi Medical Center Nanjing Medical University Wuxi 214023 China; ^3^ Anhui Province Key Laboratory of Non‐coding RNA Basic and Clinical Transformation Wannan Medical College Wuhu 241001 China; ^4^ Research Laboratory of Tumor Microenvironment Wannan Medical College Wuhu 241001 China; ^5^ Department of Thoracic Surgery Nanjing Lishui People's Hospital Zhongda Hospital Lishui Branch Southeast University Nanjing 211200 China

**Keywords:** esophageal squamous cell carcinoma, glycolysis, histone lactylation, N6‐methyladenosine, PDAI3P1

## Abstract

Esophageal squamous cell carcinoma (ESCC) is a common digestive tract malignant cancer with high incidence and mortality rate. Many studies have shown that long noncoding RNAs (lncRNAs are involved in the progression of various types of tumors. The lncRNA protein disulfide isomerase family A member 3 pseudogene 1 (PDIA3P1) promotes the progression of ESCC, but the molecular mechanism behind this is still unclear. In this study, PDIA3P1 is highly expressed in ESCC, produces more lactate by regulating glycolysis, and the increased lactate upregulates lactylation levels to drive tumor progression. Mechanistically, PDIA3P1 competes with miR‐152‐3p to prevent degradation of glucose transporter 1 (GLUT1) mRNA, and disrupts the binding between membrane‐associated RING‐CH 8 (MARCH8) and hexokinase 2 (HK2) to reduce ubiquitination degradation of HK2, thereby promoting glycolysis. High activity glycolysis produces more lactate, which upregulates the level of histone H4K8 lactylation (H4K8la) and promotes the transcription of target bone morphogenic protein 7 (BMP7). Functionally, BMP7 is involved in the regulation of ESCC progression by PDIA3P1 both in vivo and in vitro. In addition, wilms tumor 1‐associated protein (WTAP) mediated m6A modification enhances the stability of PDIA3P1 through Insulin‐like growth factor 2 mRNA‐binding protein 1 (IGF2BP1) dependent recognition. Taken together, these findings reveal the key role of PDIA3P1 regulates glycolysis‐H4K8la‐BMP7 axis in the progression of ESCC and provides new insights into the interplay between metabolic reprogramming and epigenetic regulation.

## Graphical abstract

This study reveals a new mechanism by which PDIA3P1 regulates the progression of esophageal squamous cell carcinoma (ESCC). PDIA3P1 upregulates the expression of GLUT1 and HK2, promoting glycolysis and lactate production. Lactate upregulates the lactylation level of histone H4K8 and activates the transcription of target gene BMP7, ultimately facilitating tumor progression.

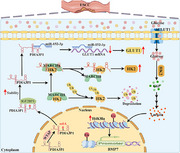

## 1 Introduction

Esophageal cancer (EC), a highly prevalent malignancy of the digestive system, ranks as the sixth leading cause of cancer‐related mortality worldwide. Pathologically, EC is classified into esophageal squamous cell carcinoma (ESCC) and esophageal adenocarcinoma (EA).^[^ ^1^ ^]^ Current therapeutic approaches primarily involve surgical resection, often in combination with radiotherapy and chemotherapy. However, patient prognosis remains poor, with a 5‐year survival rate of merely 20%.^[^ ^2^ ^]^ Unraveling the molecular mechanisms underlying ESCC progression is therefore critical for identifying novel therapeutic targets.

LncRNAs, >200 nt constitute a class of noncoding transcripts predominantly derived from genome transcription. While have limited protein‐coding capacities,^[^ ^3^ ^]^ they lncRNA exerts multiple functions through interactions with RNA, DNA, and proteins, playing important roles in gene regulation and protein function.^[^ ^4^, ^5^ ^]^ In various malignancies, lncRNAs modulate key cancer cell behaviors, including tumor cell proliferation, apoptosis, epithelial‐mesenchymal transition (EMT), invasion, metastasis, and stemness.^[^ ^6^ ^]^ For example, lncRNA CD2BP2‐DT promotes the proliferation of breast cancer cells through the YBX1/CDK1 axis.^[^ ^7^ ^]^ Linc00942 strongly promotes the expression of SOX9 by interacting with TPI1 and PKM2, thereby driving self‐renewal and TMZ resistance in GBM cells.^[^ ^8^ ^]^ In ESCC, LINC00680 functions as a competing endogenous RNA (ceRNA), regulating PAK6 expression and promoting proliferation and metastasis by sponging miR‐423‐5p.^[^ ^9^ ^]^ Similarly, our previous research demonstrated that lncRNA PDIA3P1 enhances stemness in ESCC through a positive feedback loop with OCT4. Despite its established role in ESCC progression, the precise molecular mechanisms through which PDIA3P1 drives tumor malignancy remain elusive.^[^ ^10^ ^]^

Metabolic reprogramming is a defining feature of cancer cells. Unlike normal cells, tumor cells preferentially generate energy by increasing glucose uptake and converting pyruvate into lactate, even in the presence of sufficient oxygen. This phenomenon, known as the Warburg effect, leads to excessive lactate accumulation.^[^ ^11^ ^]^ Beyond serving as a metabolic byproduct, lactate functions as a substrate for protein lysine residue modifications, playing a pivotal role in lactylation—a post‐translational modification (PTM) closely linked to tumourigenesis and progression.^[^ ^12^ ^]^ Protein lactylation is categorized into histone and non‐histone lactylation, with histone lactylation modulating gene expression by regulating downstream transcriptional activity. Notably, H3K18 lactylation has been shown to upregulate LCN2 expression,^[^ ^13^ ^]^ while H3K9 lactylation enhances LUC7L2 transcription.^[^ ^14^ ^]^ Meanwhile, histone lactylation, as an epigenetic regulatory mechanism, plays an important role in the occurrence and development of tumors. For example, H3K14la promotes the expression of downstream target NEDD4, regulates the interaction between NEDD4 and PTEN, and mediates its ubiquitination and degradation, further promoting oxaliplatin and 5‐Fu resistance in hepatocellular carcinoma.^[^ ^15^ ^]^ H4K12 lactylation upregulates GCLC expression and inhibits ferroptosis in colorectal cancer stem cells, and decreases the level of H4K12la increased the chemical sensitivity of colorectal cancer stem cells.^[^ ^16^ ^]^ Although histone lactylation has been found to regulate various tumor progression, the role of histone lactylation in ESCC remains unexplored, warranting further investigation.

N6‐methyladenosine (m6A) is the most prevalent and abundant RNA modification in eukaryotic cells, critically influencing mRNA splicing, transport, translation, degradation, and stability. As a key regulator of gene expression, m6A modification governs diverse physiological and pathological processes, including tumourigenesis and cancer progression.^[^ ^17^ ^]^ Among noncoding RNAs, lncRNAs undergo extensive m6A modification, which serves as a pivotal upstream regulatory mechanism. In nasopharyngeal carcinoma, WTAP‐mediated m6A modification of DIAPH1‐AS1 enhances its stability via IGF2BP2, thereby promoting tumourigenesis and metastasis.^[^ ^18^ ^]^ Similarly, METTL3‐mediated m6A modification facilitates OU6F2‐AS1 upregulation, driving colorectal cancer cell proliferation and lipogenesis.^[^ ^19^ ^]^ In ESCC, m6A modification also plays a pivotal role, as FTO—highly expressed in ESCC—reduces the m6A methylation of LINC00022, preventing its degradation through YTHDF2 and thereby promoting ESCC cell growth.^[^ ^20^ ^]^

This study identifies PDIA3P1 as a highly expressed lncRNA in ESCC that enhances intracellular lactate accumulation by upregulating glycolysis, thereby accelerating ESCC progression. Mechanistically, PDIA3P1 acts as a molecular sponge for miR‐152‐3p, sustaining GLUT1 expression. Concurrently, PDIA3P1 interacts with HK2, disrupting the HK2‐MARCH8 complex and preventing MARCH8‐mediated ubiquitin‐dependent degradation of HK2. The resulting upregulation of GLUT1 and HK2 augments glycolytic flux, leading to excessive lactate production. This elevated lactate level promotes histone H4K8 lactylation, which enhances BMP7 transcriptional activation and further accelerates ESCC progression both in vitro and in vivo. Additionally, WTAP‐mediated m6A modification stabilizes PDIA3P1 through interaction with the m6A reader IGF2BP1. These findings uncover a novel PDIA3P1‐driven mechanism linking metabolic reprogramming with epigenetic regulation, contributing to ESCC progression.

## 2 Results

### 2.1 PDIA3P1 Serves as a Promoter of Glycolysis in Esophageal Squamous Cell Carcinoma

Consistent with our previous research, the GEPIA 2 database, Gene Expression Omnibus (GEO) database, and our results all showed upregulation of PDIA3P1 gene expression in ESCC tissues (Figure S1A–C, Supporting Information). Especially, PDIA3P1 is highly expressed in the TNM III/IV phase and increases with increasing infiltration depth (T‐grade) (Figure S1D,E, Supporting Information). As indicated in Table S1 (Supporting Information), high PDIA3P1 expression was associated with tumor differentiation (*P*  =  0.026), TNM stage (*P*  =  0.015), and T grade (*P*  =  0.032). Moreover, PDIA3P1 expression was also significantly upregulated in human ESCC cell lines (KYSE‐30, KYSE‐150, KYSE‐520, KYSE‐410, TE‐1, and Eca‐109) compared with HEEC (Figure S1F, Supporting Information). High expression of PDIA3P1 promotes ESCC tumor progression.^[^ ^10^ ^]^ However, the underlying molecular mechanisms remain unclear. To investigate this, PDIA3P1 shRNA was transfected into TE‐1 and Eca‐109 cells via lentivirus, and stable cell lines were selected using G418 (PDIA3P1‐KD). The qRT‐PCR analysis revealed a significant reduction in PDIA3P1 expression, with PDIA3P1 3# exhibiting the most pronounced knockdown, which was subsequently used in further experiments (Figure S1G, Supporting Information). Concurrently, lentivirus to construct stable cell lines overexpressing PDIA3P1 (PDIA3P1‐OE), and the results showed that PDIA3P1 was significantly overexpressed in KYSE‐30 and KYSE‐150 cells (Figure S1H, Supporting Information).

**Figure 1:**
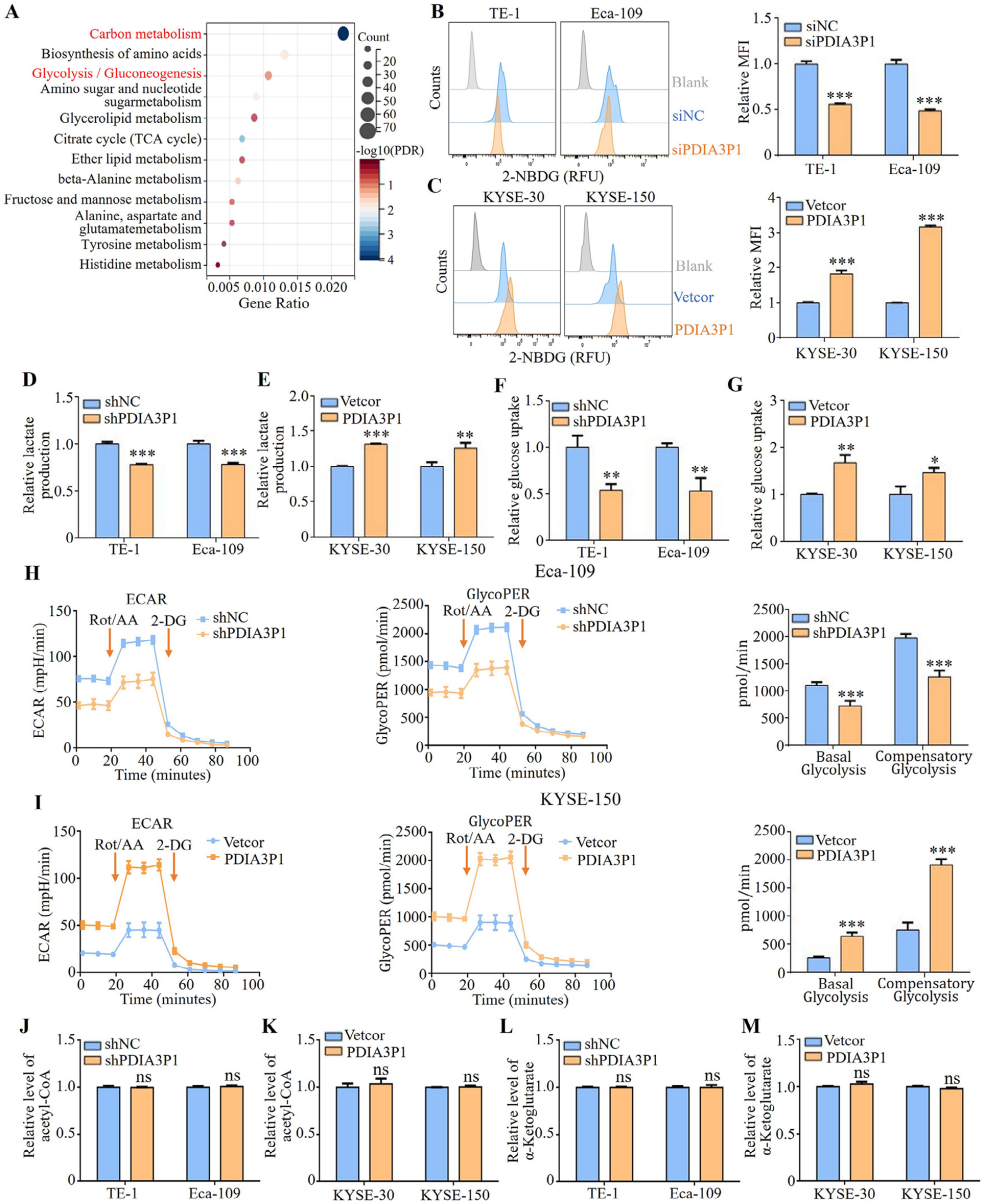
PDIA3P1 serves as a promoter of glycolysis in esophageal squamous cell carcinoma. A) KEGG metabolism analysis of the differential genes of shPDIA3P1 versus shNC by RNA‐seq. B,C) 2‐NBDG uptake was measured by flow cytometry to monitor glucose uptake in cells with transfection PDIA3P1‐siRNA (B) or PDIA3P1 expression plasmid (C) (MFI: Mean fluorescence intensity). D,E) Lactate production was measured in silenced PDIA3P1 (D) or overexpressed PDIA3P1 cells (E). F,G) Detection of glucose uptake in knocking down PDIA3P1 cells (F) or overexpressing PDIA3P1 cells (G). H,I) Seahorse metabolic analysis of ECAR, glycoPER (glycolytic proton efflux rate), basal glycolysis and compensatory glycolysis in PDIA3P1 knockdown cells (H) or PDIA3P1 overexpression cells (I) (Rot/AA: Rotenone/Antimycin A; 2‐DG: 2‐Deoxy‐D‐glucose). J,K) Relative level of acetyl‐CoA was measured in silenced PDIA3P1 cells (J) or overexpressed PDIA3P1 cells (K). L,M) Detection of relative α‐ketoglutarate levels in knocking down PDIA3P1 cells (L) or overexpressing PDIA3P1 cells (M). These data represent the mean ± S.D. of triplicates. ns: no significance; **P < 0.01; ***P < 0.001.

To explore the mechanism by which PDIA3P1 contributes to ESCC progression, RNA‐seq was conducted, comparing mRNA profiles between control cells and Eca‐109 cells with stable PDIA3P1 knockdown. Differentially expressed genes were classified via Kyoto Encyclopedia of Genes and Genomes (KEGG) pathway annotation, revealing an association between PDIA3P1 knockdown and the carbohydrate metabolism pathway (**Figure** **1**A). KEGG analysis of the TCGA database further supported the link between PDIA3P1 expression and glycolysis/gluconeogenesis (Figure S1I, Supporting Information). Based on these findings, it is hypothesized that PDIA3P1 regulates glycolysis process in ESCC cells. To verify this hypothesis, 2‐NBDG (a fluorescent D‐glucose analog) uptake, glucose uptake, and lactate production were measured. As shown in Figure 1B,C, silencing PDIA3P1 significantly inhibited 2‐NBDG uptake in TE‐1 and Eca‐109 cells, whereas PDIA3P1 overexpression enhanced 2‐NBDG uptake in KYSE‐30 and KYSE‐150 cells. Consistent with this, PDIA3P1 knockdown led to reduced lactate production (Figure 1D), while overexpression increased lactate production (Figure 1E). Moreover, PDIA3P1 knockdown reduced glucose uptake in ESCC cells (Figure 1F), whereas overexpression had the opposite effect (Figure 1G). Importantly, Seahorse glycolytic rate analysis revealed that the extracellular acidification rate (ECAR), glycoPER, basal glycolysis, and compensatory glycolysis in Eca‐109 cells with PDIA3P1 silencing were lower than in control cells (Figure 1H), while PDIA3P1 overexpression enhanced these glycolytic parameters (Figure 1I). In contrast, PDIA3P1 knockdown or overexpression did not significantly affect glucose secretion (Figure S1J,K, Supporting Information). To investigate whether the production of other metabolites (e.g., α‐ketoglutarate and acetyl‐CoA) during glucose metabolism is regulated by PDIA3P1.Neither knockdown nor overexpression of PDIA3P1 significantly regulated the intracellular levels of α‐ketoglutarate and acetyl‐CoA (Figure 1J–M). Collectively, these results demonstrate that PDIA3P1 enhances glycolysis without influencing gluconeogenesis in ESCC cells.

### 2.2 Glycolysis Mediates PDIA3P1 Regulation of Esophageal Squamous Cell Carcinoma Progression

**Figure 2:**
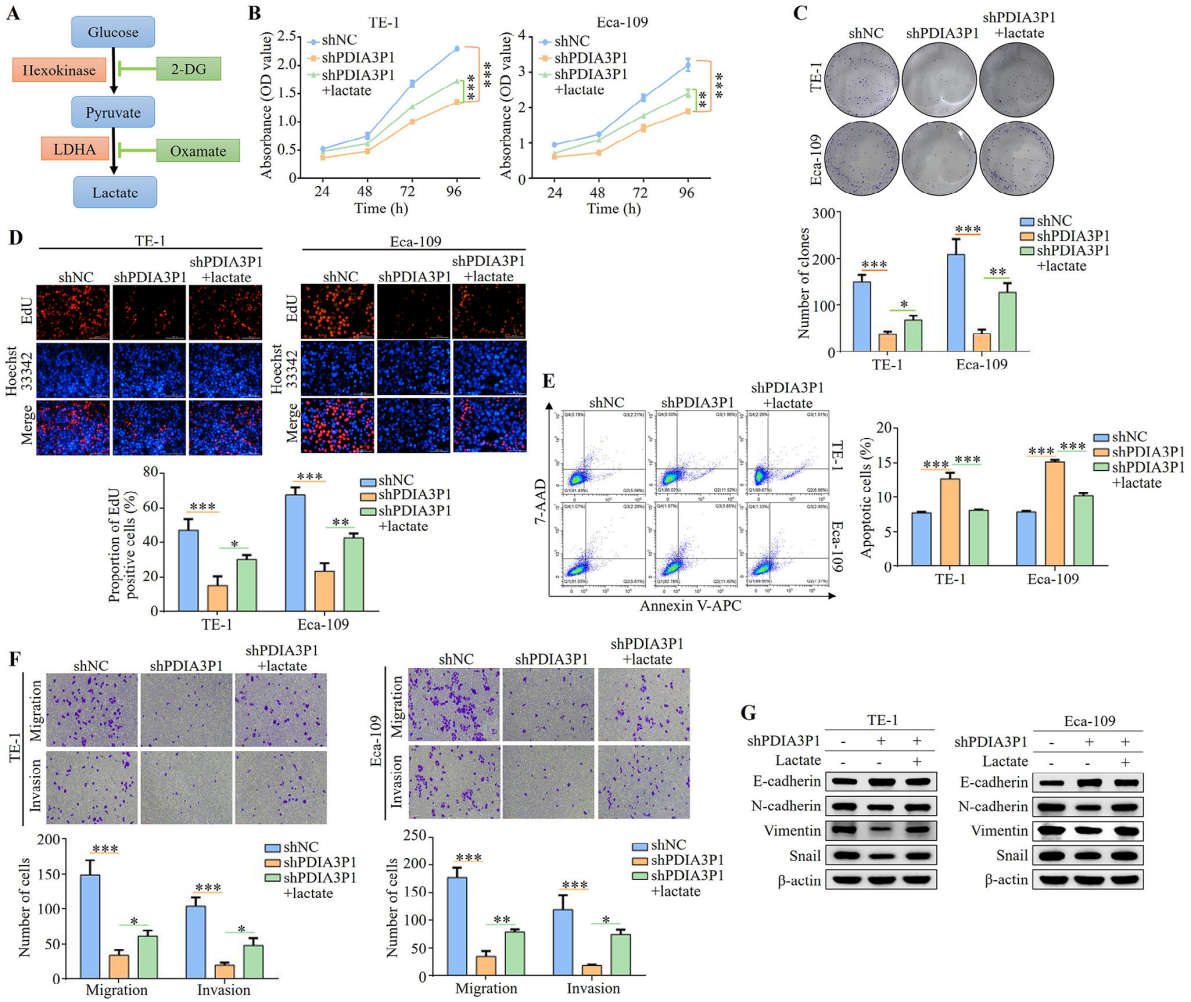
Glycolysis mediates PDIA3P1 regulation of esophageal squamous cell carcinoma progression. A) Schematic diagram of glycolysis and inhibition methods target. B–G) Stable silencing of PDIA3P1 in TE‐1 and Eca‐109 cells was treated with exogenous lactate (15mm) for 24h. B) The proliferative abilities were investigated via CCK‐8 assays. C) ESCC cell growth was analyzed using colony formation assay. The upper panel is for statistical analysis. D) EdU assays were performed to assess the proliferative ability of ESCC cells. The upper panel is for statistical analysis. Scale bar: 100 µm. E) Annexin V‐APC/7‐AAD staining for detecting apoptosis in TE‐1 and Eca‐109 cells by flow cytometry. The right panel is for statistical analysis. F) Transwell assays were used to detect changes in the cell migration and invasion. The upper panel is for statistical analysis. Transwell Scale bar: 10 µm. G) Western blot shows expression levels of E‐Cadherin, N‐Cadherin, Vimentin, and Snail. These data represent the mean ± S.D. of triplicates. *P < 0.05; **P < 0.01; ***P < 0.001.

To explore whether tumor progression is linked to PDIA3P1‐induced glycolysis and elevated lactate production in ESCC, exogenous lactate (15 mM) was added to cells with stable PDIA3P1 knockdown to counteract the lactate reduction caused by PDIA3P1 silencing and performed rescue experiments. In contrast, glycolysis inhibitors, including 2‐deoxy‐D‐glucose (2‐DG, a non‐metabolic glucose analogue) and oxamate (a lactate dehydrogenase inhibitor), were used to suppress lactate production in cells overexpressing PDIA3P1, thereby further reducing lactate levels (**Figure** **2**A). Cell proliferation was evaluated using CCK‐8 assays, colony formation assays, and EdU assays. CCK‐8 assays showed that exogenous lactate rescued the significantly impaired proliferation in TE‐1 and Eca‐109 cells resulting from PDIA3P1 silencing (Figure 2B). Knockdown of PDIA3P1 inhibited colony formation in TE‐1 and Eca‐109 cells, with partial restoration observed upon lactate supplementation (Figure 2C). Additionally, silencing PDIA3P1 inhibited DNA replication, which was partially rescued by lactate, as evidenced by EdU analysis (Figure 2D). On the contrary, 2‐DG and oxamate weakened the proliferation ability, colony formation ability, and DNA replication of KYSE‐30 and KYSE‐150 cells caused by PDIA3P1 overexpression (Figure S2A—C, Supporting Information). Apoptosis was assessed by flow cytometry, revealing that exogenous lactate reduced apoptosis levels induced by PDIA3P1 knockdown (Figure 2E). In contrast, 2‐DG and oxamate reversed the reduced apoptosis observed with PDIA3P1 overexpression (Figure S2D, Supporting Information). Transwell assays, with or without Matrigel, demonstrated that PDIA3P1 silencing decreased the migration and invasion of TE‐1 and Eca‐109 cells, and lactate treatment partially restored these abilities (Figure 2F). Treatment with 2‐DG and oxalate reduced the increased migration and invasion in ESCC cells due to stable overexpression of PDIA3P1 (Figure S2E, Supporting Information). Consistent results were obtained by Western blot analysis of EMT markers (E‐cadherin, N‐cadherin, Vimentin, and Snail) (Figure 2G; Figure S2F, Supporting Information). In conclusion, PDIA3P1 regulates the progression of ESCC by modulating glycolysis and lactate production.

### 2.3 PDIA3P1 Upregulates GLUT1 and HK2 to Promote Glycolysis in ESCC Cells

**Figure 3:**
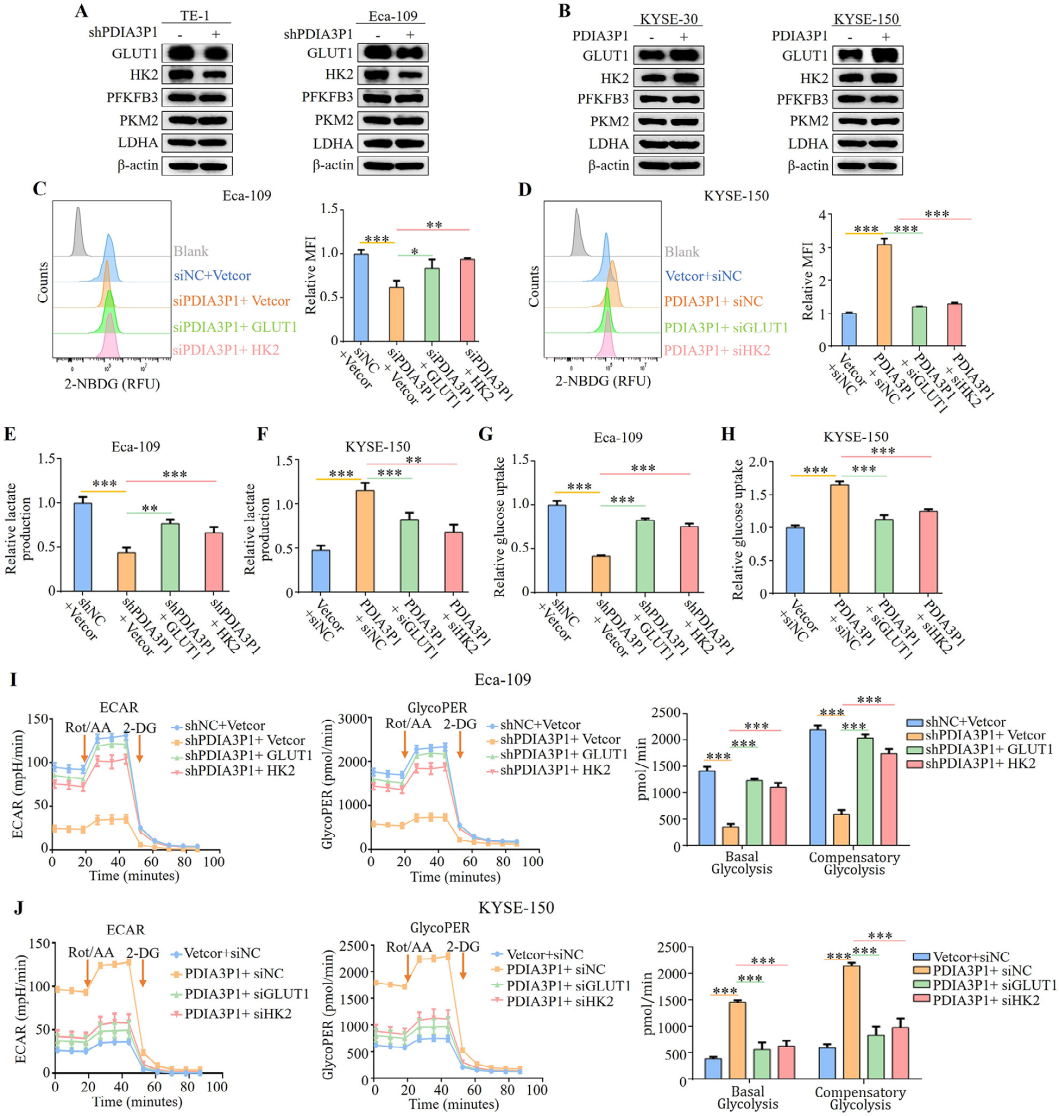
PDIA3P1 upregulates GLUT1 and HK2 to promote the glycolysis in ESCC cells. A,B) Western blot analysis of GLUT1, HK2, PFKFB3, PKM2, and LDHA after PDIA3P1 stable silencing in TE‐1 and Eca‐109 cells (A) or PDIA3P1 stable overexpressing in KYSE‐30 and KYSE‐150 cells (B). C,E,G,I) In Eca‐109 cells with stable knockdown of PDIA3P1, overexpression plasmids of GLUT1 and HK2 were transfected separately. D,F,H,J) In KYSE‐150 cells stably overexpressing PDIA3P1, GLUT1 siRNA and HK2 siRNA were transfected separately. C,D) Relative 2‐NBDG uptake detected by flow cytometry (MFI: Mean fluorescence intensity). E,F) Relative lactate production was measured using lactate assay kit. G,H) Detection of relative glucose uptake in ESCC. I,J) Cells were subjected to a Seahorse metabolic analyzer to determine the glycolytic rate including ECAR, glycoPER, basal glycolytic rate, and compensatory glycolytic rate. These data represent the mean ± S.D. of triplicates. *P < 0.05; **P < 0.01; ***P < 0.001.

To investigate how PDIA3P1 contributes to glycolysis in ESCC, the expression of five key enzymes (GLUT1, HK2, PFKFB3, PKM2, and LDHA) associated with tumor cell glycolysis was assessed by Western blot.^[^ ^21^ ^]^ The results revealed that silencing PDIA3P1 downregulated the expression of GLUT1 and HK2, while their expression was upregulated in cells with stable PDIA3P1 overexpression (**Figure** **3**A,B). This was further confirmed by IF assays in Eca‐109 cells with stable PDIA3P1 knockdown and KYSE‐150 cells with stable PDIA3P1 overexpression (Figure S3A,B, Supporting Information). Similarly, the GEPIA database results showed that GLUT1 and HK2 were highly expressed in EC, and both GEO datasets (GSE161533 and GSE11011) indicated high expression of PDIA3P1 in ESCC tissues (Figure S3C,D, Supporting Information). To determine whether PDIA3P1‐driven glycolysis depends on GLUT1 and HK2, plasmids overexpressing GLUT1 and HK2 were transfected into PDIA3P1‐knockdown Eca‐109 cells (Figure S3E, Supporting Information 1), and GLUT1 and HK2 siRNAs were transfected into KYSE‐150 cells overexpressing PDIA3P1 (Figure S3F, Supporting Information). 2‐NBDG uptake measurements showed that overexpression of GLUT1 and HK2 restored 2‐NBDG uptake in PDIA3P1 knockdown cells (Figure 3C). Conversely, transfection of GLUT1 and HK2 siRNAs in PDIA3P1‐overexpressing KYSE‐150 cells reduced 2‐NBDG uptake (Figure 3D). These results were corroborated by glucose uptake and lactate production assays (Figure 3E–H). Subsequently, metabolic parameters, including ECAR, glycoPER, basal glycolysis, and compensatory glycolysis rates, were analyzed using a Seahorse metabolic analyzer. Compared to PDIA3P1‐knockdown cells, cells co‐transfected with GLUT1 or HK2 overexpression plasmids showed upregulated glycolytic indicators in Eca‐109 cells (Figure 3I). Similarly, siGLUT1 or siHK2 transfection reduced the glycolytic promotion induced by PDIA3P1 (Figure 3J). Collectively, these results demonstrate that PDIA3P1 regulates glycolysis in ESCC cells by promoting the expression of GLUT1 and HK2.

### 2.4 PDIA3P1 Acted as a Sponge of miR‐152‐3p to Regulate GLUT1 Expression

**Figure 4:**
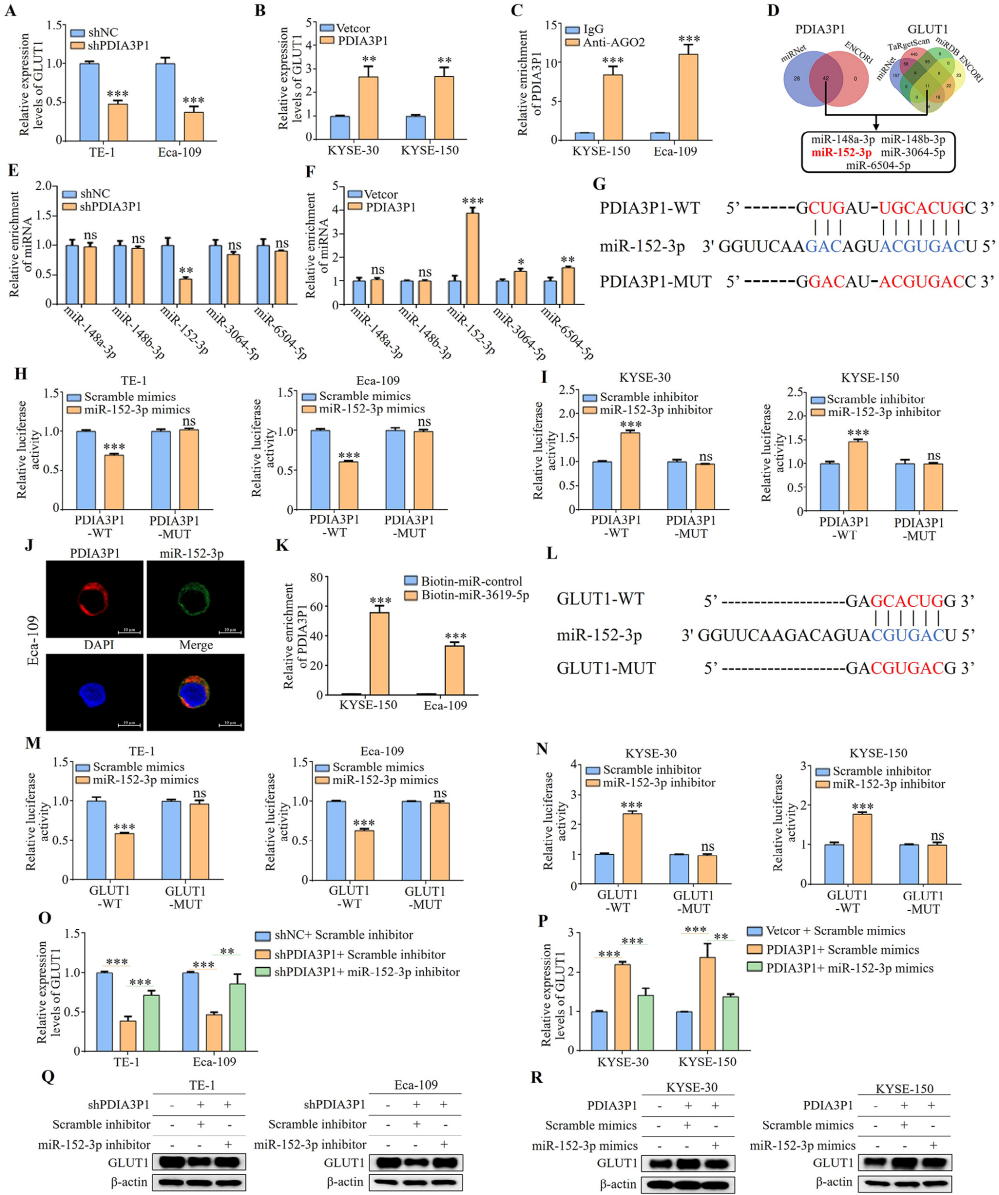
PDIA3P1 acted as a sponge of miR‐152‐3p to regulate GLUT1 expression. A,B) qRT‐PCR analyses of GLUT1 mRNA levels in stably PDIA3P1‐KD cells (A) or stably PDIA3P1‐OE cells (B). C) RIP assay for AGO2 was conducted to detect the levels of endogenous PDIA3P1 in the IP pellet of AGO2. D) The miRNAs that sponged by PDIA3P1 was predicted by miRNet and ENCORI and miRNAs that targeting GLUT1 by miRNet, TargetScan, miRDB, and ENCORI, the intersection predicted five miRNAs. E,F) AGO2‐RIP assays showed the enrichment of the predicted five miRNAs in stably PDIA3P1‐KD cells (E) or stably PDIA3P1‐OE cells (F). G) Putative binding sequence between PDIA3P1 and miR‐152‐3p. H) TE‐1 and Eca‐109 cells co‐transfected with wild‐type or mutant lncRNA PDIA3P1 and miR‐152‐3p mimics or control were detected by dual luciferase reporter assay. I) Luciferase reporters containing WT or MUT PDIA3P1 transcript were co‐transfected with miR‐152‐3p inhibitor or miR‐control in KYSE‐30 and KYSE‐150 cells. J) FISH results showing the colocalization of PDIA3P1 and miR‐152‐3p in cytoplasm of Eca‐109 cells. K) Enrichment of PDIA3P1 pulled down by biotin‐miR‐152‐3p or biotin‐miR‐control. L) Putative binding sequence of miR‐152‐3p in the 3′‐UTR of GLUT1. M) ESCC cells co‐transfected with wild‐type or mutant 3′‐UTR of GLUT1 and miR‐135b‐3p mimics or control were detected by dual luciferase reporter assay. N) Luciferase reporters containing WT or MUT 3′‐UTR of GLUT1 were co‐transfected with miR‐152‐3p inhibitor or miR‐control in ESCC cells. O) Transfection of miR‐152‐3p inhibitor into stably PDIA3P1‐KD cells, the expression levels of GLUT1 were detected by qRT‐PCR. P) Transfection of miR‐152‐3p mimics into stably PDIA3P1‐OE cells, the expression levels of GLUT1 were detected by qRT‐PCR. Q, R) Transfection of miR‐152‐3p inhibitor into stably PDIA3P1‐KD cells (Q) or miR‐152‐3p mimics into stably PDIA3P1‐OE cells (R), the expression levels of GLUT1 were detected by Western blot. These data represent the mean ± S.D. of triplicates. *P < 0.05; **P < 0.01; ***P < 0.001.

The ability of PDIA3P1 to promote glycolysis relies on the upregulation. Here, we first investigated the molecular mechanism by which PDIA3P1 regulates GLUT1 expression in ESCC cells was investigated. qRT‐PCR analysis revealed that GLUT1 mRNA levels were downregulated in stably PDIA3P1‐KD cells (**Figure** **4**A), whereas GLUT1 mRNA was upregulated in stably PDIA3P1‐OE cells (Figure 4B). PDIA3P1 has been widely reported to promote tumor progression via its ceRNA function.^[^ ^22^, ^23^, ^24^ ^]^ Previous studies also demonstrated that the subcellular localization of PDIA3P1 predominantly localizes in the cytoplasm, which provides the basic conditions for exerting ceRNA activity. RIP assays using anti‐AGO2 antibody (Figure 4C) confirmed that endogenous PDIA3P1 was preferentially enriched in AGO2‐IP complexes compared to control IgG‐IP complexes. Bioinformatics analysis including miRNet and ENCORI was used to identify miRNAs targeted by PDIA3P1, and four algorithms were employed: miRNet, Targeted Scan, miRDB, and ENCORI to predict miRNAs targeting GLUT1. The intersection of predicted miRNAs suggested that PDIA3P1 may regulate GLUT1 expression by sponging five specific miRNAs (Figure 4D). AGO2‐RIP assays indicated that miR‐152‐3p showed the most significant reduction in enrichment in stably PDIA3P1‐KD cells (Figure 4E). In contrast, miR‐152‐3p exhibited the highest enrichment in stably PDIA3P1‐OE cells compared to controls (Figure 4F). Luciferase reporter assays, using wild‐type (WT) and mutant (Mut) PDIA3P1 constructs incapable of binding to miR‐152‐3p, demonstrated that miR‐152‐3p mimics significantly reduced luciferase activity in cells transfected with WT PDIA3P1, but did not affect mutant PDIA3P1 constructs (Figure 4G,H). Conversely, a miR‐152‐3p inhibitor enhanced luciferase activity in WT PDIA3P1, but had no effect on mutant PDIA3P1 (Figure 4I). Fluorescence in situ hybridization (FISH) assays further confirmed the colocalization of PDIA3P1 and miR‐152‐3p in the cytoplasm of ESCC cells (Figure 4J). Subsequently, biotin‐labeled miRNA pull‐down assays showed significantly increased PDIA3P1 interaction in the ESCC cells with biotin‐labeled miR‐152‐3p compared to biotin‐labeled control (Figure 4K).

To further validate the role of miR‐152‐3p in the regulation of GLUT1 by PDIA3P1, a wild‐type GLUT1 3'‐UTR vector and a mutant vector unable to bind miR‐152‐3p were constructed (Figure 4L). Luciferase reporter assays revealed that miR‐152‐3p mimics significantly reduced the luciferase activity of the WT GLUT1 3′‐UTR, but had no effect on the MUT GLUT1 3′‐UTR (Figure 4M). Conversely, miR‐152‐3p inhibitors exhibited opposite effects in both KYSE‐30 and KYSE‐150 cells (Figure 4N). Western blot and qRT‐PCR analyses confirmed that miR‐152‐3p inhibitors significantly reduced both mRNA and protein levels of GLUT1 in stably PDIA3P1‐KD cells, while miR‐152‐3p mimics showed the opposite trend in stably PDIA3P1‐OE cells (Figure 4O–R). These results collectively demonstrate that PDIA3P1 promotes GLUT1 expression via miR‐152‐3p, establishing a novel ceRNA network involving PDIA3P1, miR‐152‐3p, and GLUT1 in ESCC.

### 2.5 PDIA3P1 Repressed HK2 Degradation via MARCH8 Mediated Ubiquitin–Proteasome Pathway

**Figure 5:**
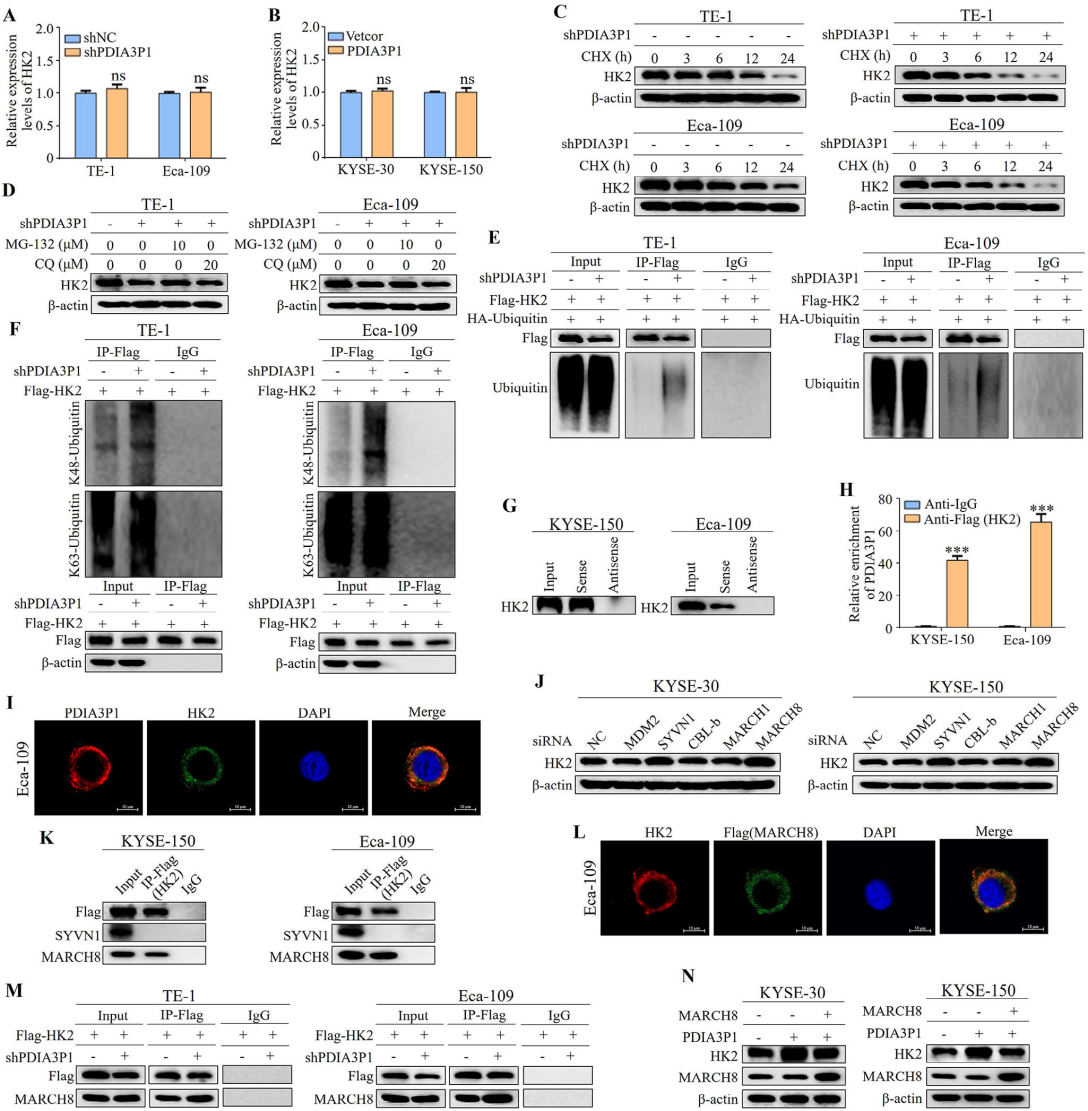
PDIA3P1 repressed HK2 degradation via MARCH8 mediated ubiquitin–proteasome pathway. A, B) qRT‐PCR analyses of HK2 mRNA levels in stably PDIA3P1‐KD cells (A) or stably PDIA3P1‐OE cells (B). C) Western blot analysis of HK2 protein stability using CHX (200 µg/mL) when PDIA3P1 was knocked down. D) Western blot analysis expression of HK2 after treatment with MG132 (10 µM) or CQ (20 µM) in PDIA3P1‐KD cells. E) The ubiquitination level of HK2 was detected by Western blot in cells that stabilized silenced PDIA3P1. IgG was used as a negative control. F) PDIA3P1‐KD cells were co‐immunoprecipitated with Flag antibody, and the expression of K48‐linked ubiquitin and K63‐linked ubiquitin in the immune complex was detected. G) PDIA3P1 pull‐down followed by Western blot validated its interaction with HK2. H) Transfection of Flag‐HK2 plasmid into cells, RIP assay was performed using the HK2 antibody in KYSE‐150 and Eca‐109 cells. I) FISH and IF analysis showing the colocalization of PDIA3P1 and HK2 in cytoplasm. J) After knocking down SYVN1, MARCH1, MARCH8, MDM2, and CBL‐b in cells, respectively, the expression of HK2 was detected by Western blot. K) Interactions between SYVN1 or MARCH8 and HK2 in gastric cancer cells were verified via Co‐IP assays. L) IF analysis showing the colocalization of MARCH8 and HK2 in cytoplasm. M) Transfection of Flag‐HK2 plasmid into cells, co‐IP with anti‐flag antibody for detecting the interaction of MARCH8 and HK2 in the PDIA3P1‐KD cells. N) Transfection of MARCH8 cDNA plasmid into PDIA3P1‐OE cells and detection of HK2 expression by Western blot. These data represent the mean ± S.D. of triplicates. ns: no significance; ***P < 0.001.

To elucidate the mechanism by which PDIA3P1 promotes HK2 expression in ESCC, HK2 mRNA levels in cells with PDIA3P1 knockdown or overexpression were analyzed using qRT‐PCR. However, no significant differences in HK2 mRNA expression were observed between the experimental and control cells (**Figure** **5**A,B). This led us to hypothesize that PDIA3P1 regulates HK2 expression by enhancing its protein stability. To test this, cells were treated with the protein synthesis inhibitor cycloheximide (CHX) for various time points to assess HK2 protein stability. The results indicated that HK2 had a shorter half‐life in PDIA3P1‐KD cells and a longer half‐life in PDIA3P1‐OE cells (Figure 5C; Figure S4A, Supporting Information). Two major pathways regulate protein degradation: the ubiquitin‐proteasome and autophagy‐lysosome pathways.^[^ ^25^ ^]^ Cells were treated with the autophagy inhibitor chloroquine (CQ) and the proteasome inhibitor MG‐132. Western blot analysis revealed that the reduction in HK2 levels caused by PDIA3P1 knockdown could be reversed by MG‐132 treatment (Figure 5D), while PDIA3P1 overexpression in the presence of MG‐132 further upregulated HK2 expression (Figure S4B, Supporting Information). No such effect was observed following CQ treatment. These results suggest that the ubiquitin‐proteasome pathway is likely involved in PDIA3P1‐mediated stabilization of HK2. To confirm this, the impact of PDIA3P1 on HK2 ubiquitination was assessed. Co‐IP assays demonstrated that PDIA3P1 knockdown significantly increased the ubiquitination of HK2 (Figure 5E), while PDIA3P1 overexpression reduced HK2 ubiquitination (Figure S4C, Supporting Information). Next, we determined the specific ubiquitin chain type mediating PDIA3P1‐dependent HK2 ubiquitination. HK2 has been confirmed to produce K48‐linked ubiquitination and K63‐linked ubiquitination.^[^ ^26^, ^27^, ^28^ ^]^ Here, the Co‐IP results showed that PDIA3P1 knockdown increased K48‐linked ubiquitination of HK2, whereas PDIA3P1 overexpression suppressed it. In addition, PDIA3P1 has no significant regulatory effect on the levels of K63‐linked ubiquitination of HK2 (Figure 5F; Figure S4D, Supporting Information).

The interaction between PDIA3P1 and HK2 likely mediates the regulation of HK2 ubiquitination by PDIA3P1. RPISeq analysis, which predicts RNA‐protein interactions based solely on sequence data, suggested the possibility of such an interaction between PDIA3P1 and HK2 (Figure S4E, Supporting Information). To verify this, Western blot analysis was conducted on protein complexes extracted from PDIA3P1 pulldown assays. The results confirmed that HK2 specifically binds to the sense sequence of PDIA3P1, but not to the antisense sequence (Figure 5G). Consistent with this, RIP assays showed that PDIA3P1 was significantly enriched in RNA‐protein complexes precipitated with anti‐Flag antibodies in Flag‐HK2‐transfected cells, compared to the IgG control group (Figure 5H). FISH and IF analysis further demonstrated the interaction between PDIA3P1 and HK2 in the cytoplasm (Figure 5I). To explore the mechanism by which PDIA3P1 inhibits the ubiquitin‐proteasome degradation of HK2, UbiBrowser was used to predict potential E3 ligases that might interact with HK2 (Figure S4F, Supporting Information). Among the top five predicted E3 ligases (SYVN1, MARCH1, MARCH8, MDM2, and CBL‐b), silencing of MDM2, CBL‐b, and MARCH1 did not reduce HK2 expression (Figure 5J; Figure S4G, Supporting Information). However, silencing of SYVN1 and MARCH8 resulted in a decrease in HK2 levels. To confirm the involvement of SYVN1 and MARCH8 as E3 ligases of HK2, Flag‐HK2 was overexpressed in KYSE‐150 and Eca‐109 cells, followed by immunoprecipitation (IP) with anti‐Flag and IgG antibodies. Notably, only MARCH8 was co‐precipitated with HK2 (Figure 5K). Moreover, IF experiments confirmed the colocalization of HK2 and MARCH8 (Figure 5L). These results establish that MARCH8 is an E3 ubiquitin ligase for HK2. Given that PDIA3P1 inhibits HK2 ubiquitination and MARCH8 is identified as the E3 ligase of HK2, whether PDIA3P1 modulates the interaction between HK2 and MARCH8 was examined. Co‐IP assays showed that in PDIA3P1‐KD cells, Flag‐HK2 precipitated significantly more MARCH8 compared to control cells (Figure 5M). Conversely, in PDIA3P1‐OE cells, less MARCH8 was precipitated with Flag‐HK2 compared to controls (Figure S4H, Supporting Information). Finally, overexpression of MARCH8 in PDIA3P1‐OE cells partially reversed the increased expression of HK2 (Figure 5N), suggesting that PDIA3P1 stabilizes HK2 by disrupting the interaction between HK2 and MARCH8 in ESCC.

### 2.6 PDIA3P1 Increases H4K8la Level by Promoting Glycolysis in ESCC Cells

**Figure 6:**
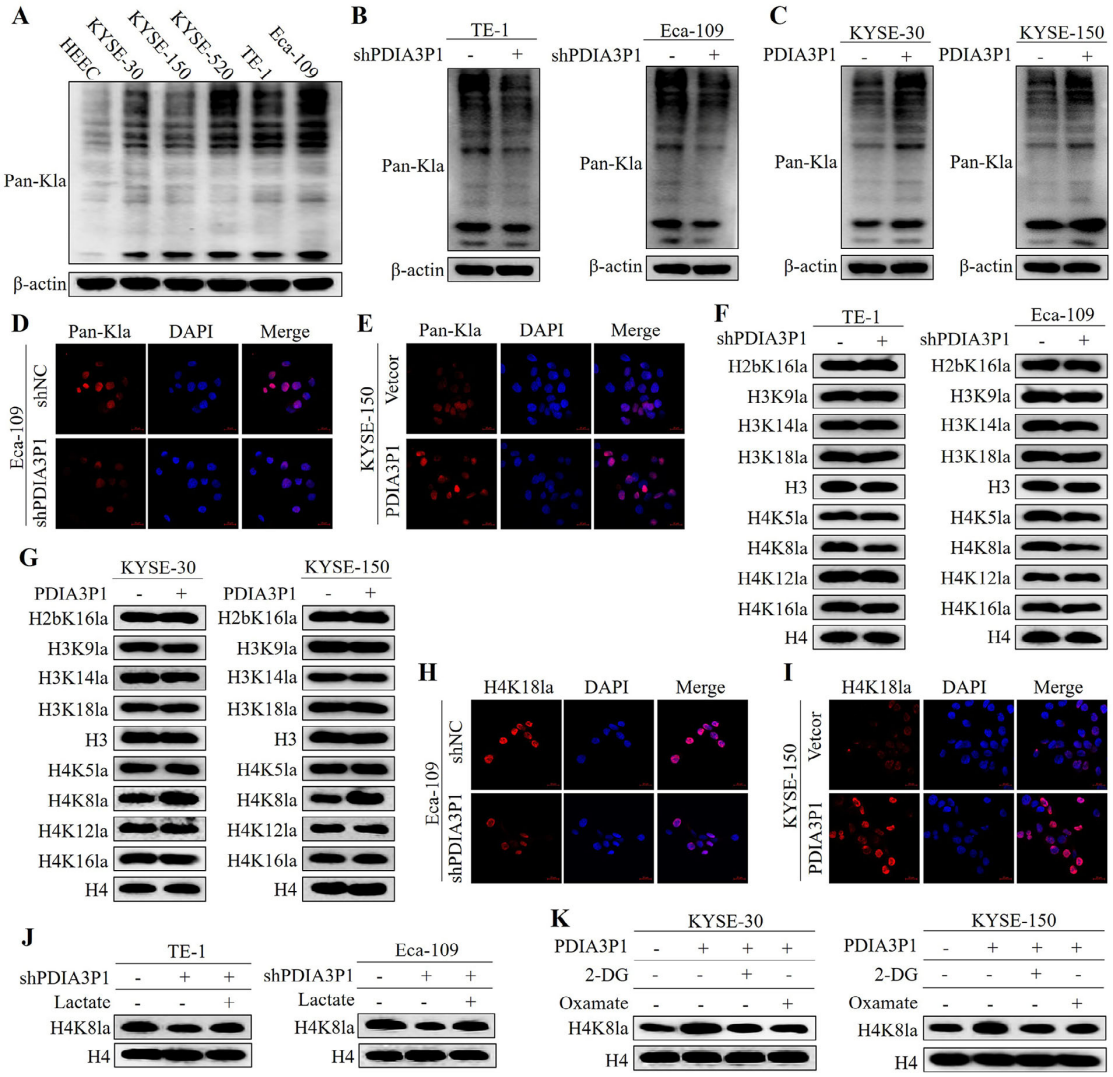
PDIA3P1 increases H4K8la level by promoted glycolysis in ESCC cells. A) Western blot shows the levels of Pan Kla in normal cell line HEEC and five ESCC cell lines (KYSE‐30, KYSE‐150, KYSE‐520, TE‐1, and Eca‐109). B,C) Western blot analysis of Pan‐ Kla after PDIA3P1 stable silencing in TE‐1 and Eca‐109 cells (B) or PDIA3P1 stable overexpressing in KYSE‐30 and KYSE‐150 cells (C). D,E) Representative images of IF staining revealing the effect of PDIA3P1 knockdown in Eca‐109 (D) or PDIA3P1 overexpressed in KYSE‐150 (E) on the expression of Pan‐ Kla. F, G) Western blot analysis of site‐specific histone lactylation in PDIA3P1‐KD cells (F) or PDIA3P1‐OE cells (G). H, I) Representative images of IF staining revealing the effect of PDIA3P1 knockdown in Eca‐109 (H) or PDIA3P1 overexpressed in KYSE‐150 (I) on the expression of H4K8la. J) PDIA3P1‐KD cells were treated with lactate for 24 h, Western blot measured the level of H4K8 lactylation. K) H4K18la levels were detected in PDIA3P1‐OE cells cultured in 2‐DG or oxamate for 24 h by western blot.

Considering the impact of PDIA3P1 on glycolysis and lactate production, PDIA3P1 may promote ESCC progression via histone lactylation. As expected, Western blot assays in **Figure** **6**A revealed increased global lactylation levels in ESCC cell lines. Knockdown of PDIA3P1 significantly reduced global lactylation in TE‐1 and Eca‐109 cells (Figure 6B), while overexpression of PDIA3P1 elevated lactylation levels in KYSE‐30 and KYSE‐150 cells (Figure 6C). Immunofluorescence staining further confirmed that global lactylation levels were decreased in PDIA3P1‐KD cells and increased in PDIA3P1‐OE cells (Figure 6D,E). Histone lactylation, a key aspect of lactylation modification, plays an increasingly critical role in tumor cells. Using antibodies targeting various forms of histone lactylation, Western blot analysis revealed that PDIA3P1 most significantly regulated H4K8la levels in ESCC cells (Figure 6F,G). Immunofluorescence also showed that PDIA3P1 regulated H4K8la levels (Figure 6H,I). To explore the role of glycolysis in PDIA3P1‐mediated histone lactylation, stable PDIA3P1‐KD cells were treated with exogenous lactate. The results showed that exogenous lactate reversed the reduction of H4K8la levels caused by PDIA3P1 knockdown (Figure 6J). In contrast, treatment with glycolysis inhibitors (2‐DG and Oxamate) in PDIA3P1‐OE cells partially restored the H4K8la levels that were elevated by PDIA3P1 overexpression (Figure 6K). Together, these results indicate that histone lactylation, particularly H4K8la, is regulated by PDIA3P1 and contributes to the promotion of ESCC progression.

### 2.7 BMP7 is a Target of H4K8 Lactylation in ESCC Cells

**Figure 7:**
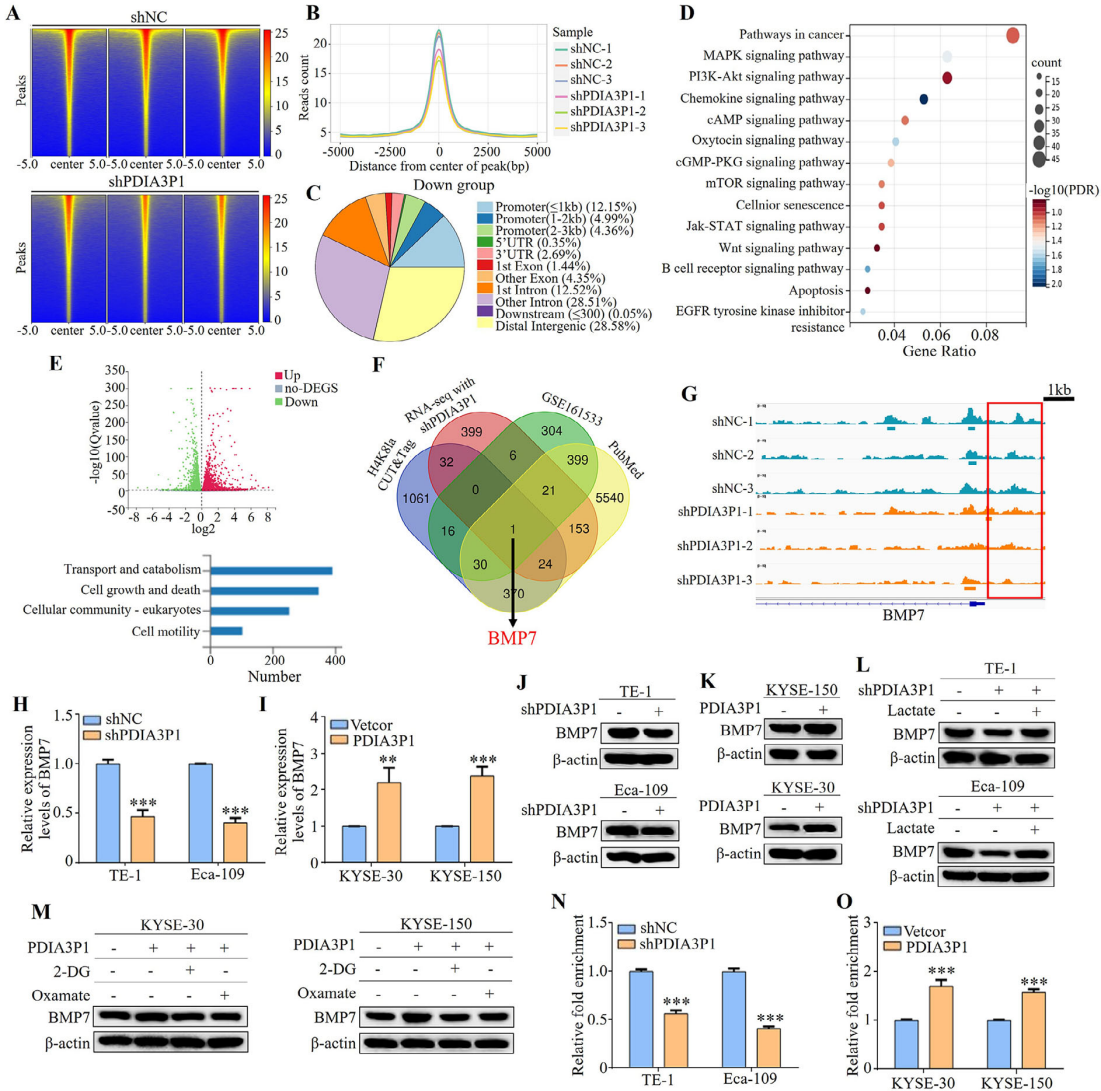
BMP7 is a target of H4K8 lactylation in ESCC cells. A) The binding density of H4K8la was visualized by deepTools: the heatmap illustrates the CUT&Tag tag counts on the various H4K8la enrichment peaks in shNC and shPDIA3P1 cells. B) Distribution of H4K8la sites relative to translation start site (TSS). C Genome‐wide distribution of downregulated H4K8la‐binding peaks in shPDIA3P cells. D) KEGG analysis of the decreased H4K8la binding peaks at candidate target genes. E) Transcriptome sequencing was performed in control and shPDIA3P1 cells. F) Venn Diagram of CUT&Tag, RNA‐seq, GEO, and pubmed database to identify the potential downstream targets of H4K8la. G) Integrative Genomics Viewer tracks of CUT&Tag showing enriched H4K8la in the promotors of BMP7. The red rectangles indicate the peak regions of H4K8la on target‐gene promoters. H‐K) BMP7 mRNA and protein levels were measured in PDIA3P1‐KD cells (H, J) or PDIA3P1‐OE cells (I, K). L) Western blotting analysis of BMP7 expression in PDIA3P1‐KD cells cultured in lactate for 24 h. M) Western blot analysis of BMP7 expression in PDIA3P1‐OE cells cultured in 2‐DG or oxamate for 24 h. N, O) Using antibodies against H4K8la, CUT&Tag‐qPCR analysis for binding status at the BMP7 promotor of PDIA3P1‐KD cells (N) or PDIA3P1‐OE cells (O). These data represent the mean ± S.D. of triplicates. **P < 0.01; ***P < 0.001.

Histone lactylation is a novel epigenetic modification known to directly regulate the transcriptional activation of target genes, thereby influencing tumor progression.^[^ ^29^ ^]^ Next, we attempt to determine how H4K8la participates in the regulation of PDIA3P1 on ESCC progression. Therefore, we performed whole genome CUT&Tag analysis using anti‐H4K8la antibodies in Eca‐109 cells to identify potential genes regulated by H4K8la in the cells (Dataset S1 and Figure S5A, Supporting Information). The results revealed a significant reduction in the enrichment of H4K8la in PDIA3P1‐KD cells compared to control cells (**Figure** **7**A), with a marked decrease observed in the transcription start site (TSS) region, showing an ≈21.5% reduction in enrichment at the promoter region (Figure 7B,C). KEGG and Gene Ontology (GO) analyses of the downregulated peak genes in the promoter region of PDIA3P1‐KD cells indicated that these genes are involved in multiple signaling pathways related to tumor progression (Figure 7D; Figure S5B, Supporting Information). Additionally, RNA sequencing analysis was performed to identify differentially expressed genes between control cells and PDIA3P1‐KD cells (Figure 7E; Dataset S2, Supporting Information). To pinpoint the target genes activated by H4K8la, this study overlapped the gene sets with the downregulated promoter peaks from CUT&Tag, reduced expression levels from RNA sequencing, genes highly expressed in ESCC tissues from the GEO dataset (GSE161533), and genes associated with ESCC progression in the PubMed database. This analysis led to the identification of BMP7 as a key target gene (Figure 7F). To confirm that PDIA3P1 activates BMP7 transcription via H4K8la, the H4K8la peak at the BMP7 promoter region was analyzed using Integrative Genomics Viewer, which showed a significant decrease in the H4K8la signal at this position in PDIA3P1‐KD cells (Figure 7G). qRT‐PCR results confirmed that BMP7 mRNA levels were downregulated in PDIA3P1‐KD TE‐1 and Eca‐109 cells (Figure 7H), while overexpression of PDIA3P1 led to BMP7 upregulation in KYSE‐30 and KYSE‐150 cells (Figure 7I). Consistent results were obtained from Western blot and IF analyses (Figure 7J,K; Figure S5C,D, Supporting Information). When cells were treated with exogenous lactate, the decrease in BMP7 expression caused by PDIA3P1 silencing was reversed (Figure 7L). Conversely, glycolysis inhibitors (2‐DG and oxamate) reduced BMP7 protein levels in PDIA3P1‐overexpressing cells (Figure 7M). Finally, CUT&Tag‐qPCR analysis further confirmed the enrichment of H4K8la at the BMP7 promoter region (Figure S5E, Supporting Information). In PDIA3P1‐KD cells, H4K8la enrichment was significantly reduced, while in PDIA3P1‐OE cells, it was enhanced (Figure 7N,O). These results collectively suggest that BMP7 transcription is positively regulated by H4K8la and that this pathway may play a critical role in PDIA3P1‐mediated ESCC progression.

### 2.8 PDIA3P1 Promotes Tumourigenesis of Esophageal Squamous Cell Carcinoma Through BMP7 both in Vitro and in Vivo

**Figure 8:**
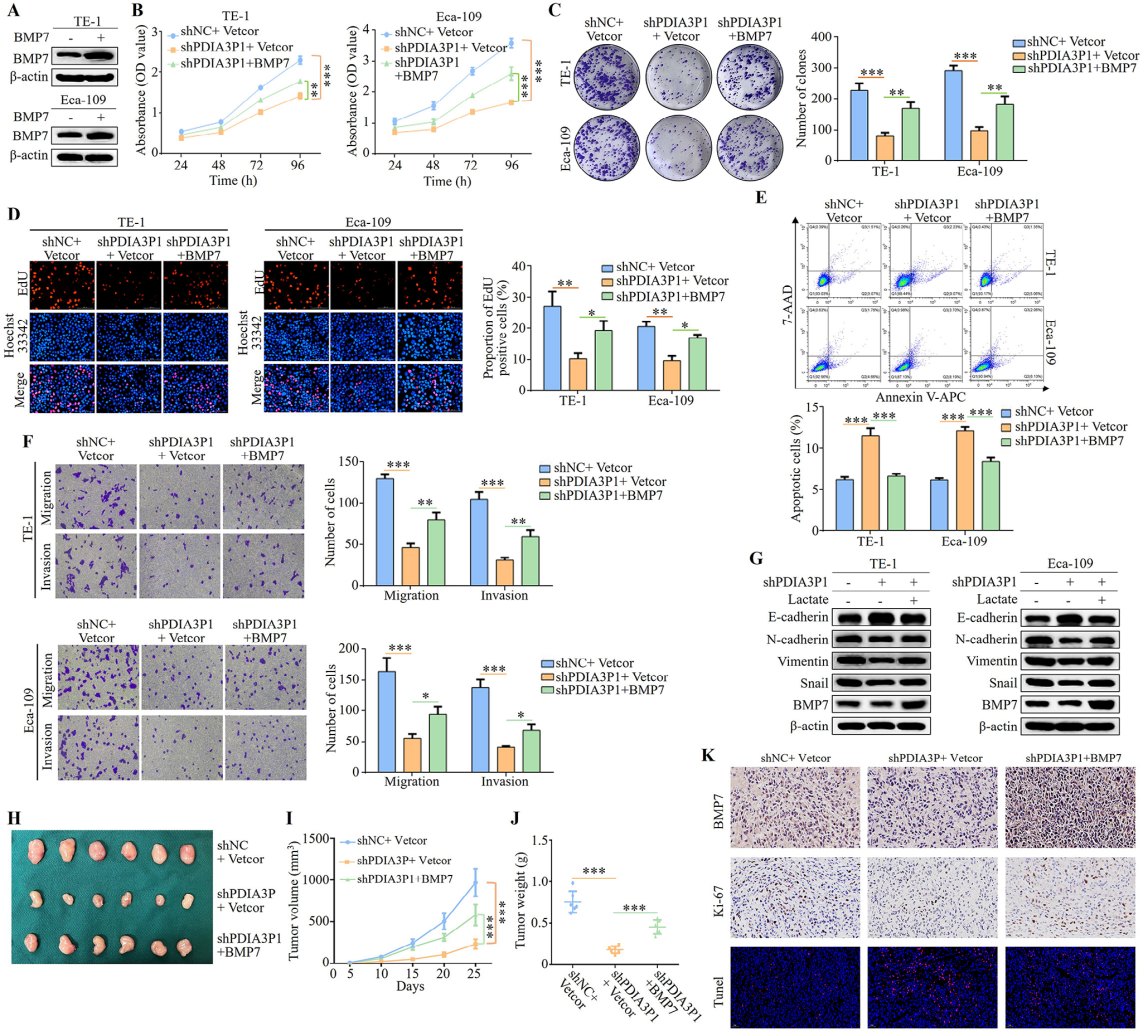
PDIA3P1 promotes tumorigenesis of esophageal squamous cell carcinoma through BMP7 both in vitro and in vivo. A) Western blot detection of BMP7 expression to reflect the effect of transfection of BMP7 by lentivirus. B‐G) Using lentivirus to construct BMP7 stable overexpression cell lines in TE‐1 and Eca‐109 cells with stably PDIA3P1‐KD. B) Proliferation of cells was analyzed using CCK8 assay. C) Tumor growth of TE‐1 and Eca‐109 cells was evaluated by colony formation assay. D) The proliferative abilities of ESCC cells were investigated using EdU assays. EdU scale bar: 100 µm. E) The flow cytometry showing apoptosis of cells by Annexin V‐APC and 7‐AAD staining. F) The migration and invasion ability was assessed by transwell assays. Transwell scale bar: 10 µm. G) Western blot shows expression levels of E‐Cadherin, N‐Cadherin, Vimentin, Snail, and BMP7. H‐K) Eca‐109 cells transfected with control, shPDIA3P1 or shPDIA3P1 and BMP7 were injected subcutaneously to establish a tumorigenesis model in nude mice (*n* = 6). H) Photograph and comparison of tumor sizes in the indicated groups. I) The parameters of subcutaneous tumors were measured and recorded every 5 days, and calculated the volume of the tumor according to the formula below: tumor volume  =  0.5 × length × width × width (mm3). J) Tumor weights in the indicated groups. K) Ki‐67 and BMP7 expression of representative IHC of nude mice tumor tissues. Scale bar, 20 µm. IF staining of TUNEL in subcutaneous tumor tissue sections. Scale bar: 50 µm. These data represent the mean ± S.D. of triplicates. *P < 0.05; **P < 0.01; ***P < 0.001.

To explore the involvement of BMP7 in the promotion of ESCC progression by PDIA3P1, BMP7 was transfected into PDIA3P1‐KD cells using lentiviral vectors to achieve overexpression (**Figure** **8**A). Subsequently, cell proliferation, apoptosis, invasion, and metastasis were assessed in vitro, and tumor growth ability was evaluated in vivo. CCK‐8, colony formation, and EdU assays revealed that BMP7 overexpression restored the proliferative capacity of ESCC cells suppressed by PDIA3P1 silencing (Figure 8B–D). Flow cytometry analysis further demonstrated that BMP7 overexpression reduced apoptosis in stably PDIA3P1‐KD cells (Figure 8E). Transwell assays showed that BMP7 transfection reversed the invasion and migration abilities of ESCC cells inhibited by PDIA3P1 knockdown (Figure 8F). Western blot analysis of EMT markers indicated that the protein levels of N‐cadherin, Vimentin, and Snail were elevated in the group co‐transfected with shPDIA3P1 and BMP7 cDNA compared to the shPDIA3P1‐only group, while E‐cadherin expression was reduced (Figure 8G). These results demonstrate that BMP7 is involved in PDIA3P1‐mediated regulation of ESCC progression in vitro.

To further assess the role of BMP7 in regulating ESCC tumor growth in vivo, PDIA3P1‐KD and BMP7‐overexpressing cells (or control cells) were subcutaneously transplanted into nude mice, establishing a xenograft tumor model. After 25 days, tumor growth was monitored, and the results showed that BMP7 overexpression significantly accelerated tumor growth, as indicated by increased tumor volume and weight compared to PDIA3P1 knockdown alone (Figure 8H‐J). IHC staining for Ki‐67 and TUNEL further confirmed the increased proliferative capacity and reduced apoptosis in tumor tissues from BMP7‐overexpressing mice (Figure 8K). In summary, both in vitro and in vivo studies highlight the critical role of BMP7 in PDIA3P1‐mediated promotion of ESCC progression.

### 2.9 Increased Stability of PDIA3P1 is via IGF2BP1 Recognition of WTAP‐Mediated m6A Modification

**Figure 9:**
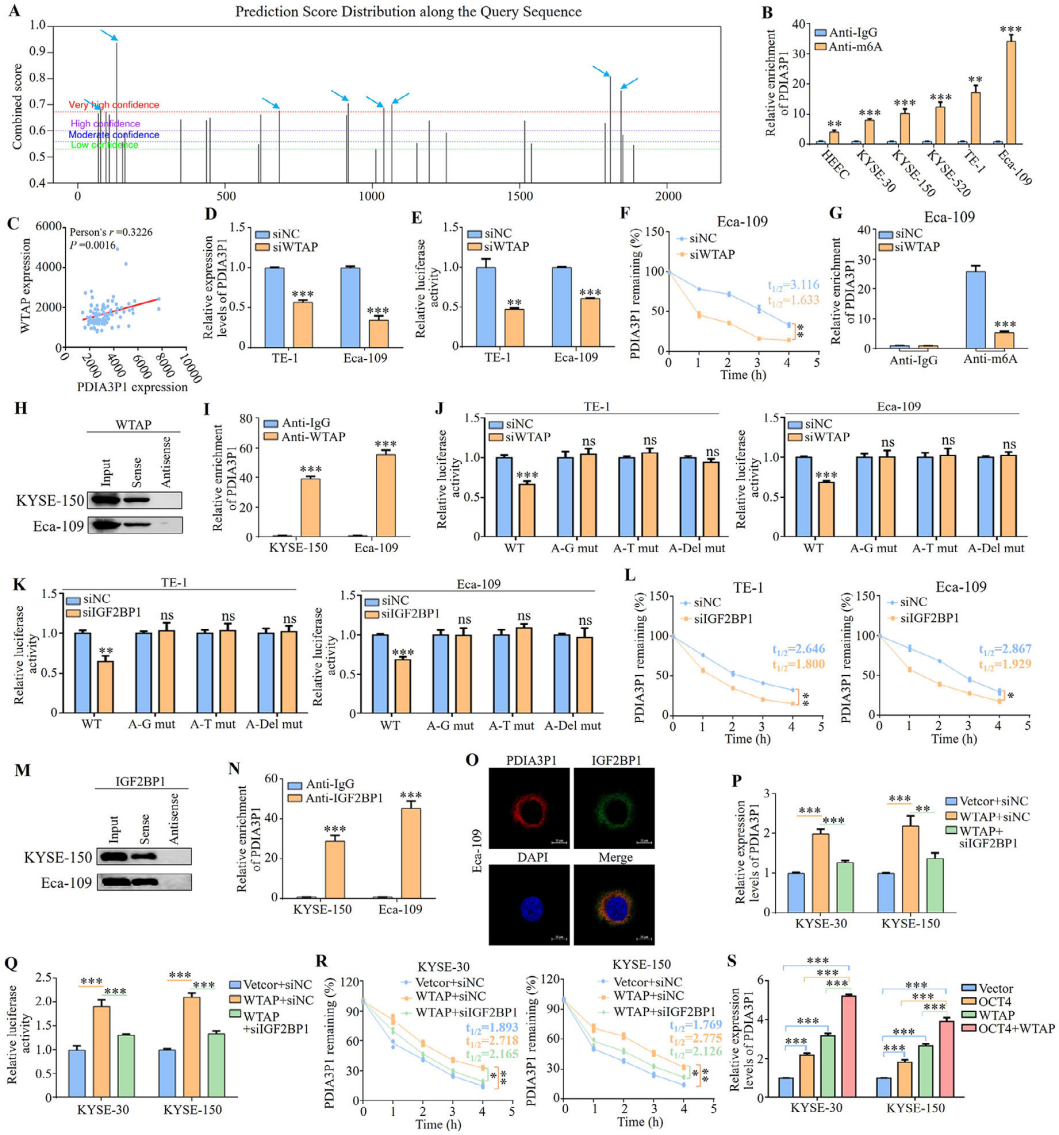
Increased stability of PDIA3P1 is via IGF2BP1 recognition of WTAP‐mediated m6A modification. A) The enriched and specific m6A peak distribution of PDIA3P1 predicted by SRAMP. B) MeRIP‐qPCR analysis of m6A modification level of PDIA3P1 in normal cell line HEEC and five ESCC cell lines (KYSE‐30, KYSE‐150, KYSE‐520, TE‐1, and Eca‐109). C) Correlation analysis showing a positive correlation between WTAP and PDIA3P1 expression. D) qRT‐PCR analysis of PDIA3P1 expression in TE‐1 and Eca‐109 cells with WTAP knockdown. E) Relative luciferase activity in TE‐1 and Eca‐109 cells co‐transfected with luciferase reporter pmirGLO‐PDIA3P1 and WTAP siRNA. F) WTAP silencing cells treated with actinomycin D (10 µg mL^−1^) for the various time points; the level of PDIA3P1 was examined by qRT‐PCR. G) The m6A modification level of PDIA3P1 was examined in WTAP‐silencing cells by MeRIP‐qPCR. H) PDIA3P1 pull‐down followed by Western blot validated its interaction with WTAP. I) RIP assay was performed using the WTAP antibody in KYSE‐150 and Eca‐109 cells. J) Relative luciferase activity of the wild‐type and its mutants pmirGLO‐PDIA3P1 reporter vectors when transfected with WTAP siRNA in TE‐1 and Eca‐109 cells (WT: wild‐type; A–G mut: adenine residues substituted by guanine; A–T mut: adenine residues substituted by thymine; A–Del mut: adenine residues deleted). K) Relative luciferase activity of the wild‐type and its mutants pmirGLO‐PDIA3P1 reporter vectors during IGF2BP1 silencing in ESCC cells. L) PDIA3P1 stability in control and IGF2BP1‐silenced cells, the level of PDIA3P1 was examined by qRT‐PCR. M) PDIA3P1 pull‐down followed by Western blot validated its interaction with IGF2BP1. N) RIP assay was performed using the IGF2BP1 antibody. O) FISH and IF double staining showing the co‐localization of PDIA3P1 and IGF2BP1 in the cytoplasm. P) qRT‐PCR analysis of PDIA3P1 expression in KYSE‐30 and KYSE‐150 cells co‐transfected with WTAP overexpression vector and the IGF2BP1 siRNA. Q) Relative luciferase activity in KYSE‐30 and KYSE‐150 cells co‐transfected with WTAP overexpression vector and the IGF2BP1 siRNA. R) PDIA3P1 stability in ESCC cells co‐transfected with WTAP overexpression vector and the IGF2BP2 siRNA, the level of PDIA3P1 was examined by qRT‐PCR. S) Transfection or co‐transfection of OCT4 cDNA or WTAP cDNA separately, and expression of PDIA3P1 was detected by qRT‐PCR. These data represent the mean ± S.D. of triplicates. ns: no significance; *P < 0.05; **P < 0.01; ***P < 0.001.

In our previous findings, PDIA3P1 was highly expressed in ESCC and is a direct target of transcription factor OCT4. Epigenetic regulation plays a pivotal role in gene expression, with m6A modification being significantly involved in the modulation of lncRNA expression.^[^ ^30^, ^31^ ^]^ In Tan's research, the oncogene PDIA3P1 was identified as an m6A‐related pseudogene, exhibiting substantial m6A peaks and elevated m6A levels in head and neck squamous cell carcinoma.^[^ ^24^ ^]^ Meanwhile, SRAMP prediction showed that PDIA3P1 enriched multiple specific m6A peaks (**Figure** **9**A). This suggests that PDIA3P1 may undergo m6A modification in ESCC. To test this, MeRIP‐qPCR results demonstrated increased m6A levels of PDIA3P1 in ESCC cell lines (KYSE‐30, KYSE‐150, KYSE‐520, TE‐1, and Eca‐109) compared to the normal oesophageal epithelial cell line HEEC, indicating that m6A contributes to the upregulation of PDIA3P1 expression (Figure 9B). Further analysis of the TCGA database revealed a positive correlation between PDIA3P1 expression and the m6A enzyme WTAP, with no such correlation observed for other m6A enzymes (METTL3, METTL14, FTO, ALKBH5) (Figure 9C; Figure S6A, Supporting Information). Additionally, WTAP expression was found to be elevated in ESCC tissues (Figure S6B, Supporting Information). Silencing WTAP in TE‐1 and Eca‐109 cells via siRNA (Figure S6C, Supporting Information) and overexpressing WTAP in KYSE‐30 and KYSE‐150 cells (Figure S6D, Supporting Information) led to a decrease and increase in PDIA3P1 expression, respectively, as confirmed by qRT‐PCR (Figure 9D; Figure S6E, Supporting Information). Dual luciferase reporter assays further demonstrated that WTAP knockdown significantly reduced luciferase activity of a PDIA3P1 reporter (Figure 9E), while WTAP overexpression enhanced reporter activity (Figure S6F, Supporting Information). Consistent with these findings, treatment with actinomycin D to block transcription revealed that siRNA‐mediated WTAP knockdown shortened the half‐life of PDIA3P1 in Eca‐109 cells (Figure 9F), whereas WTAP overexpression prolonged its half‐life (Figure S6G, Supporting Information). MeRIP‐qPCR analysis confirmed that WTAP knockdown reduced m6A levels of PDIA3P1 in Eca‐109 cells (Figure 9G), while WTAP overexpression elevated m6A levels of PDIA3P1 in KYSE‐150 cells (Figure S6H, Supporting Information). Finally, RNA pulldown with the PDIA3P1 sense sequence and RIP assays with WTAP antibody demonstrated a direct interaction between PDIA3P1 and WTAP (Figure 9H,I). To further investigate the specific functional sites of PDIA3P1 regulated by WTAP in an m6A dependent manner. As indicated by the arrow in Figure 9A, PDIA3P1 has 8 m6A sites (RRACH motif) with high confidence. Next, we constructed three mutant PDIA3P1 sequences and cloned them into the dual luciferase reporter construct pmirGLO, in which the adenine residues in predicted m6A motifs of PDIA3P1 were substituted by guanine (A–G mut), thymine (A–T mut), or deleted (Del‐mut), respectively. Dual luciferase reporter assays demonstrated that the regulatory effect of WTAP on the activity of PDIA3P1 luciferase was eliminated after the m6A residue mutation (Figure 9J; Figure S6I, Supporting Information). These results collectively indicate that WTAP modulates the stability of PDIA3P1 in ESCC cells via m6A modification.

To identify the m6A reader responsible for interpreting the WTAP‐mediated m6A modification of PDIA3P1, insulin‐like growth factor 2 mRNA‐binding proteins (IGF2BPs; including IGF2BP1, IGF2BP2, and IGF2BP3), well‐established RNA‐binding proteins that stabilize their target RNAs,^[^ ^32^ ^]^ were examined. IGF2BPs might be involved in the m6A‐dependent stabilization of PDIA3P1. Consistent with this, siRNA‐mediated knockdown of IGF2BP1 in cells resulted in a decrease in PDIA3P1 expression, whereas silencing IGF2BP2 and IGF2BP3 did not produce a similar effect (Figure S6J,K, Supporting Information). Dual luciferase reporter assays further corroborated this finding (Figure S6L, Supporting Information). Similarly, IGF2BP1 had no effect on the luciferase activity of PDIA3P1 constructs with three m6A residue mutations (Figure 9K). More importantly, PDIA3P1 degradation was accelerated in IGF2BP1‐silenced TE‐1 and Eca‐109 cells (Figure 9L). To assess whether IGF2BP1 directly interacts with PDIA3P1, a pulldown experiment was conducted, and IGF2BP1 was detected in the pulled‐down protein by Western blot (Figure 9M). RIP assays using an anti‐IGF2BP2 antibody also confirmed significant enrichment of PDIA3P1 (Figure 9N). Furthermore, FISH combined with immunofluorescence revealed co‐localization of PDIA3P1 and IGF2BP1 in the cytoplasm (Figure 9O).

To further confirm the necessity of IGF2BP1 for the stability of PDIA3P1 promoted by WTAP‐mediated m6A modification, IGF2BP1 siRNA was co‐transfected into KYSE‐30 and KYSE‐150 cells that were transfected with WTAP cDNA. As shown in Figure 9P,Q, WTAP overexpression increased both PDIA3P1 expression and luciferase activity of the PDIA3P1 reporter, and this effect was reversed by IGF2BP1 knockdown. Similar results were obtained from half‐life assays of PDIA3P1 (Figure 9R). As previously established, OCT4 can promote PDIA3P1 transcription. To investigate the relationship between OCT4 and WTAP in regulating PDIA3P1 expression, we found no mutual regulatory effect between OCT4 and WTAP (Figure S6M,N, Supporting Information). qRT‐PCR results showed that overexpression of either OCT4 or WTAP individually promoted PDIA3P1 expression, and their combined overexpression had a synergistic effect. Notably, WTAP exhibited a stronger upregulatory effect on PDIA3P1 expression than OCT4 when overexpressed individually (Figure 9S). These data collectively suggest that WTAP‐mediated m6A modification enhances PDIA3P1 expression through IGF2BP1‐dependent RNA stability.

## 3 Discussion

EC, a prevalent malignant tumor of the digestive tract, ranks sixth in global mortality and seventh in incidence.^[^ ^33^ ^]^ The insidious onset and rapid progression of ESCC contribute to the poor prognosis of most patients. Therefore, investigating the molecular mechanisms underlying ESCC progression is crucial. Recent studies have highlighted the significant role of lncRNAs in the development of various malignancies, including ESCC. Transcriptomic analyses have revealed numerous dysregulated lncRNAs in ESCC tissues. For instance, LncRNA CASC9 is overexpressed and exerts oncogenic effects by recruiting EZH2 and subsequently altering H3K27me3 levels to negatively regulate PDCD4 expression.^[^ ^34^ ^]^ LINC00941 is upregulated in ESCC, exacerbating tumor progression by forming a positive feedback loop involving LINC00941‐ILF2/YBX1‐SOX2.^[^ ^35^ ^]^ Similarly, our previous research identified that PDIA3P1 is overexpressed in ESCC and promotes tumor progression, but the underlying mechanisms remain unexplored.^[^ ^10^ ^]^

**Figure 10:**
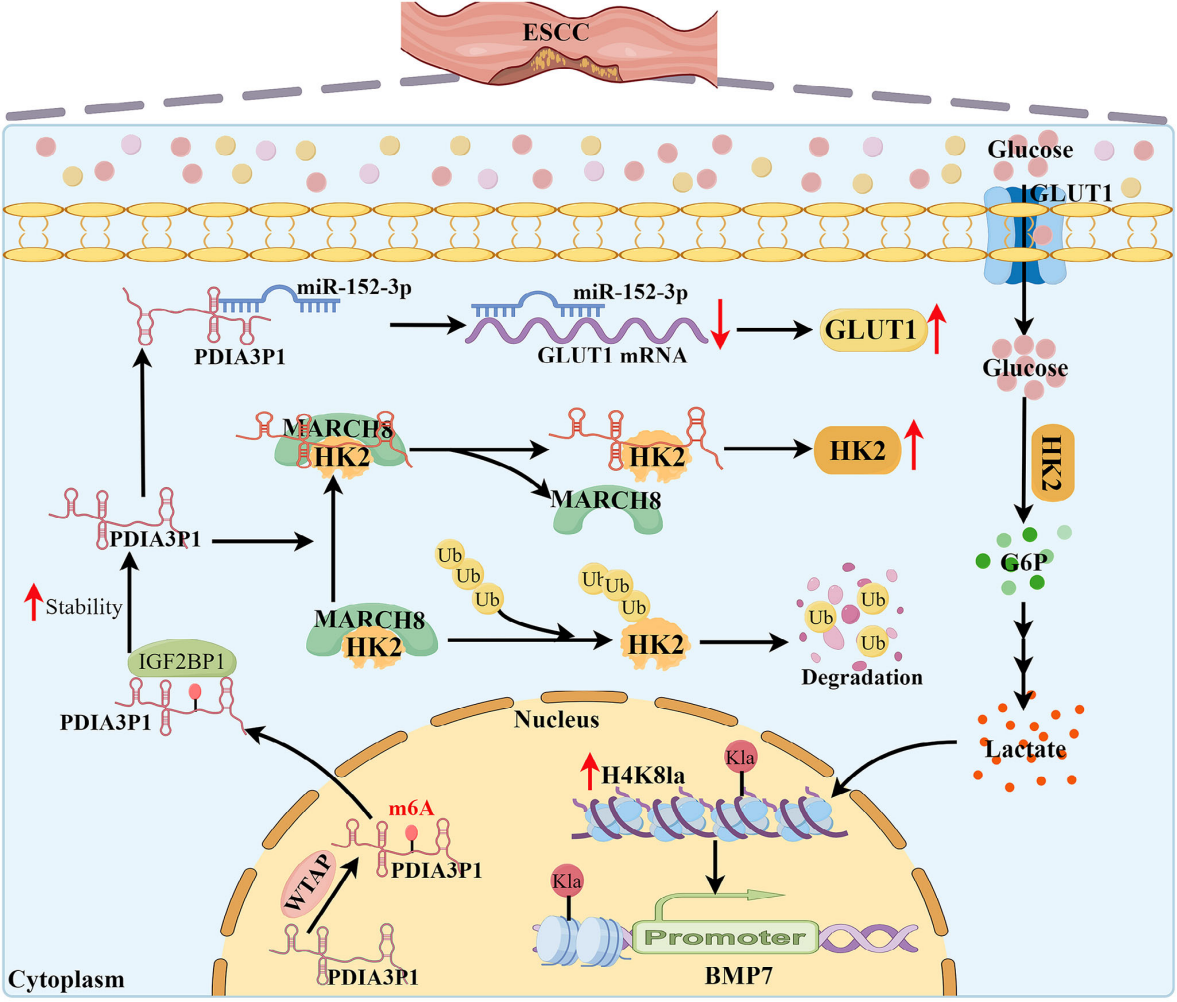
Schematic representation of the study.

In this study, high PDIA3P1 expression accelerated ESCC progression by enhancing glycolysis, increasing lactate production, and inducing BMP7 expression. Mechanistically, PDIA3P1 upregulates GLUT1 expression by functioning as a ceRNA for miR‐152‐3p. Additionally, PDIA3P1 interacts with HK2, disrupting the HK2‐MARCH8 complex and enhancing HK2 expression. These two pathways collectively elevate glycolysis and lactate production. Elevated intracellular lactate levels increase histone H4K8la, promoting the transcription of BMP7. Finally, this study explored the mechanism underlying the high expression of PDIA3P1 and found that WTAP‐mediated m6A modification stabilizes PDIA3P1 through the IGF2BP1‐dependent pathway. Our findings integrate lncRNA biology, cellular metabolism, histone modifications, and RNA modification regulation, offering new insights into ESCC progression and suggesting that PDIA3P1 could serve as a novel therapeutic target for ESCC (**Figure** **10**).

Cellular metabolic reprogramming is a key factor supporting various malignant behaviours in tumor cells, with aerobic glycolysis being a hallmark of cancer.^[^ ^11^, ^36^ ^]^ In tumor cells, glucose metabolism is significantly elevated—up to ten times higher than in non‐tumor cells—resulting in markedly increased lactate levels. Aerobic glycolysis not only provides a rapid ATP source^[^ ^37^ ^]^ but also influences multiple tumor processes, including modulating the tumor microenvironment and regulating signal transduction pathways.^[^ ^38^, ^39^ ^]^ Numerous studies have confirmed that lncRNAs are involved in tumor metabolic reprogramming, particularly in glycolysis. For example, LINC00520 promotes glycolysis in osteosarcoma by upregulating ENO1 protein expression via blocking FBXW7‐mediated ENO1 ubiquitination and proteasomal degradation.^[^ ^40^ ^]^ In gastric cancer, the interaction between lncRNA CCAT1 and splicing factor PTBP1 inhibits its degradation via ubiquitination, facilitating the transition from PKM1 to PKM2, thereby enhancing glycolysis.^[^ ^41^ ^]^ In the present study, PDIA3P1 promoted glycolysis in ESCC by upregulating the expression of GLUT1 and HK2. GLUT1, a glucose transporter, plays a pivotal role in tumor metabolism and significantly accelerates cancer cell metabolism under both aerobic and anaerobic conditions.^[^ ^42^ ^]^ HK2, as the rate‐limiting enzyme in glycolysis, is directly involved in the metabolic reprogramming of tumor cells.^[^ ^43^ ^]^ Our research identified two mechanisms by which lncRNAs regulate GLUT1 and HK2 expression. First, lncRNAs can act as ceRNAs, “sponging” complementary miRNAs to promote the disinhibition of target molecules.^[^ ^44^ ^]^ Previous studies have demonstrated the regulation of GLUT1 expression via the ceRNA mechanism. For instance, lncRNA TMPO‐AS1 upregulates GLUT1 by directly binding to miR‐140 and miR‐143 in endometrial cancer,^[^ ^45^ ^]^ while lncRNA MCM3AP‐AS1 acts as a competitive endogenous RNA for miR‐218, enhancing GLUT1 expression in papillary thyroid cancer.^[^ ^46^ ^]^ Additionally, lncRNA PVT1 binds to miR‐150‐5p, reducing its inhibition of GLUT1 and promoting its expression.^[^ ^47^ ^]^ The present study identified a novel ceRNA mechanism whereby PDIA3P1 acts as a ceRNA for miR‐152‐3p, reducing the degradation of GLUT1 mRNA by miR‐152‐3p. Meanwhile, miR‐152‐3p is a miRNA associated with DNA methylation. Previous studies have shown that miR‐152‐3p affects the DNA methylation level of NF2 by regulating DNMT1 expression.^[^ ^48^ ^]^ Our study found that miR‐152‐3p regulates GLUT1 expression, thereby influencing lactate production. Similarly, lactate has been confirmed to directly regulate DNMT1 expression as a signaling molecule.^[^ ^38^ ^]^ Here, we speculate that lactate can also regulate DNA methylation levels through an indirect regulation, such as by promoting histone lactylation and regulating the transcription of DNA methylation‐related enzymes (e.g., DNMT1, DNMT3A, DNMT3B, and DNMT3L), thereby increasing intracellular DNA methylation levels. This establishes a tight link between miR‐152‐3p and histone lactylation in regulating intracellular DNA methylation levels. Beyond ceRNA activity, lncRNAs can also function as scaffold molecules, regulating protein interactions and stability. While most research on lncRNA‐mediated regulation of HK2 has focused on the ceRNA mechanism, some studies have explored direct modulation of HK2 expression. For instance, lncRNA DLEU2 binds to miR‐455 to induce HK2 expression,^[^ ^49^ ^]^ lncRNA DLGAP1‐AS2 upregulates HK2 by binding to miR‐411a,^[^ ^50^ ^]^ and lncRNA CASC7 promotes HK2 expression by acting as a sponge for miR‐143‐3p.^[^ ^51^ ^]^ In the present study, a novel mechanism by which lncRNA regulates HK2 was revealed at the protein stability level. The interaction between PDIA3P1 and HK2 reduces the binding of HK2 to its E3 ubiquitin ligase MARCH8, thereby decreasing the ubiquitination and degradation of HK2. Our research provides new insights into the direct role of lncRNAs in regulating glycolysis.

PTMs of histones play a critical role in regulating gene expression, with numerous modifications occurring at the N‐terminal tail of histones.^[^ ^52^ ^]^ Recently, lactylation has emerged as a novel histone modification. Several lactylation sites have been identified, including H3K9la, H3K18la, H4K5la, and H4K12la.^[^ ^53^ ^]^ Increased histone lactylation in the promoter region has been shown to activate gene expression. For example, histone H3K18la enhances METTL3 transcription while mediating RNA m6A modification to promote the immunosuppressive effect of tumor‐infiltrating myeloid cells.^[^ ^54^ ^]^ H3K18la also promotes the expression of the autophagy‐enhancing protein RUBCNL in colorectal cancer, increasing resistance to bevacizumab treatment,^[^ ^55^ ^]^ and regulates the transcription of YTHDF2, which plays a role in ocular melanoma progression.^[^ ^56^ ^]^ Additionally, histone H3K9la has been implicated in conferring temozolomide resistance to glioblastoma through LUC7L2.^[^ ^14^ ^]^ Histone lactylation is a product of lactate generated by cellular metabolism. In this study, PDIA3P1 induced increased lactate production in cells, leading to alterations in global lactylation and histone lactylation. Further investigation revealed that PDIA3P1 influences ESCC progression by regulating histone H4K8la. Data from techniques such as CUT&Tag and RNA‐seq demonstrated that H4K8la is strongly enriched in the BMP7 promoter region, promoting BMP7 transcription, which correlates with PDIA3P1 expression. Our findings provide new insights into the interplay between cancer metabolic reprogramming and epigenetic regulation, offering novel directions for studying ESCC progression.

Post transcriptional modifications of RNA, including m6A, N1‐methyladenosine (m1A), and 5‐methylcytosine (m5C), are essential for regulating transcriptional stability, translation, alternative splicing, and subcellular localization.^[^ ^57^ ^]^ Among these, m6A is the most extensively studied RNA modification. LncRNAs, as key regulatory elements in human cells, also undergo m6A modification.^[^ ^58^ ^]^ M6A is a reversible, dynamically regulated process facilitated by methyltransferases (“writers”) and reversed by demethylases (“erasers”), with its effects on RNA mediated by distinct m6A binding proteins (“readers”).^[^ ^59^ ^]^ WTAP, a critical component of the m6A methyltransferase complex, catalyzes m6A methylation on RNA. Previous studies have demonstrated that WTAP plays a pivotal role in m6A modification of lncRNAs, such as enhancing the stability of DIAPH1‐AS1 in nasopharyngeal carcinoma, thereby promoting tumor growth and metastasis.^[^ ^18^ ^]^ Additionally, WTAP‐mediated m6A modification of lncRNA NORAD contributes to intervertebral disc degeneration^[^ ^60^ ^]^ and promotes osteosarcoma progression by stabilizing FOXD2‐AS1.^[^ ^61^ ^]^ In the context of ESCC, WTAP‐mediated alterations in m6A modification levels of PDIA3P1 were found to enhance its stability. As part of the m6A “reader” family, IGF2BP1/2/3 regulates lncRNA stability in an m6A‐dependent manner.^[^ ^32^ ^]^ Identifying specific m6A readers is crucial for understanding the full impact of WTAP‐mediated m6A modification. This study reveals that WTAP‐mediated m6A modification enhances PDIA3P1 expression through IGF2BP1‐dependent RNA stabilization.

In summary, the current study elucidated that glycolysis and histone lactylation mediated the regulatory effects of PDIA3P1 on ESCC progression. Meanwhile, m6A modification is involved in the stability regulation of PDIA3P1. This work proposes a novel molecular mechanism underlying ESCC progression regulated by lncRNAs, offering new promising molecular targets and potential therapeutic strategies for ESCC treatment.

## 4 Experimental Section

### Tissues Collection

Thirty three pairs of ESCC and matched normal tissue samples were collected from ESCC patients who underwent surgery from May 2023 to December 2024 in The First Affiliated Hospital of Wannan Medical College. This study was approved by the Institutional Ethics Committee of the The First Affiliated Hospital of Wannan Medical College (Approval Number: 2024–66). Written informed consent was obtained from each subject. The methods were carried out in accordance with the approved guidelines.

### Cell Culture and Inhibitor

Human normal esophageal epithelial cell line HEEC and HEK293T cells were cultured in DMEM medium (Gibco, USA). KYSE‐30, KYSE‐150, KYSE‐410, KYSE‐520, TE‐1, and Eca109 (human esophageal squamous cell carcinoma) were cultured in RPMI 1640 medium. All medium were supplemented with 10% fetal bovine serum (FBS) (BDBIO, China) and 1x Penicillin‐Streptomycin Solution (BDBIO, China). Cells were maintained at 37 °C in a 5% CO2 incubator. All cell lines provided by immocell (IMMOCELL, China). MG‐132 (HY‐13259), Cycloheximide (CHX, HY‐12320), Actinomycin D (HY‐17559), Chloroquine (CQ, HY‐17589A), 2‐DeoXy‐D‐glucose (HY‐13966) and Oxamic acid sodium (HY‐W013032A) were purchased from MedChemExpress. Lactate was purchased from Sigma.

### SiRNA, Plasmid and Lentivirus Transduction

The cells (30–50% density) were transfected with 100nm siRNA mixed with 4.5 µL GenMute (SL100568, SignaGen) in a cell culture plate. Cells were cultured for 48 h before plating for protein or RNA isolation and various assays. Sequences of siRNAs are listed in Table S2 (Supporting Information). For plasmid, cells were transfected with PolyJet DNA transfection (SL100688, SignaGen) reagent when the cell density reached 80–90% confluence. All plasmids used in the article are sourced from MiaoLingBio (Wuhan, China). For lentivirus transduction, recombinant lentiviral particles were produced by transient co‐transfection of PDIA3P1 plasmids into HEK293T cells. The shRNAs recombinant lentiviral particles of PDIA3P1 are derived from GenePharma. Sequences of shRNAs are listed in Table S3 (Supporting Information). Stably PDIA3P1‐overexpression (OE) cells were selected using 2 µg mL^−1^ puromycin (HY‐B1743, MCE). Stably PDIA3P1‐knockdown (KD) ESCC cells were selected using 500µg mL^−1^ G‐418 (HY‐K1056, MCE). In these lentiviral transducted cells, the expression levels of PDIA3P1 were examined by qRT‐PCR.

### RNA Extraction and Quantitative Real‐Time PCR Analysis

Total RNA was prepared from the various treated cells using Eastep Super Total RNA Extraction Kit (LS1040, Promega) according to the manufacturer's instructions. Each RNA sample was then reverse transcribed into cDNAs using GoScript Reverse Transcription Mix, Oligo(dT) (A2791, Promega). cDNA and appropriate primers were plated in a 96‐well plate and gene expression levels were measured using GoTaq qPCR Master Mix (A6001, Promega) with a Bio‐Rad CXF96 PCR system (Hercules, CA, USA). For miRNA, reverse transcription using miRNA 1st Strand cDNA Synthesis Kit (by stem loop) (MR101‐01, Vazyme), qPCR using miRNA Unimodal SYBR qPCR Master Mix (MQ101‐02, Vazyme). Each sample was repeated three times. 2−ΔΔCt method was used to quantify the relative gene expression level, with β‐actin as the reference gene. All qRT‐PCR Primer sequences are shown in Table S4 (Supporting Information). All primers used for miRNAs reverse transcription are shown in Table S5 (Supporting Information).

### Glycolytic Process Evaluation

Here, 2‐NBDG uptake, glucose uptake, and lactate production are used to evaluate the status of glycolysis. Cells were treated overnight with glucose free culture medium and then exposed to 2‐NBDG (a fluorescent derivative of glucose) (N13195, Invitrogen) for 60 min. 2‐NBDG can be excited by 488 nm laser and fluorescence recorded using flow cytometry. The supernatants were then measured for their lactate production using a Lactate‐Glo Assay (J5021, Promega) according to the manufacturer's instructions. The Glucose Uptake was measured using a Glucose Uptake‐Glo Assay (J1341, Promega) according to the manufacturer's instructions.

### Seahorse Metabolic Analyzer Glycolytic Rate Assay

Forty thousand cells were seeded into each well in XF‐24 cell culture and incubated overnight. The next day, the cells were rinsed with Seahorse detection buffer. The glycolytic rate was determined using Seahorse XF Glycolytic Rate Assay Kit (103344‐100) with Seahorse XF‐24 Extracellular Flux Analyzer (Agilent Seahorse Bioscience, USA) according to the manufacturer's instruction. The analyzer injected Rot/AA and 2‐DG (2‐deoxy‐glucose) automatically. The extracellular acidification rate (ECAR), glycolytic proton efflux rate (glycoPER), basal glycolysis rate, and compensatory glycolysis rate were analyzed using Seahorse XF24 software.

### Glucose Secretion Assay

Cell medium was replaced with RPMI 1640 containing 0.1% serum for 16 h. Cells were washed twice with PBS to remove glucose and then incubated for 6 h in glucose production assay medium (glucose and phenol red‐free RPMI 1640 containing 2 mmol L^−1^ sodium pyruvate, 20 mmol L^−1^ sodium lactate, 2 mmol L^−1^ l‐glutamine and 15 mmol L^−1^ HEPES). Medium (200 µL) was sampled for measurement of glucose concentration. According to the instructions of the reagent supplier, use the glucose content detection kit (GOD‐POD, microplate method) (60408ES60, Yeasen) in a 96 well plate.

### Detection of Acetyl‐CoA and α‐Ketoglutarate Levels

For the cell samples, the cells were resuspended in Extraction Buffer, subjected to ultrasonic disruption on ice, and then centrifuged at 12 000×g for 10 min at 4 °C. Finally, the supernatant was collected and placed on ice for testing. The acetyl‐CoA levels were assessed using an Acetyl Coenzyme A (Acetyl‐CoA) Assay Kit (KTB1260, Abbkine) according to the manufacturer's instructions. The α‐ketoglutarate levels were assessed using a α‐Ketoglutaric Acid (α‐KG) Content Assay Kit (AKAC016M, Beijing Boxbio Science & Technology) according to the manufacturer's instructions.CCK‐8 assay

Cell viability was assessed using the CCK‐8 assay kit. The transfected cells were diluted to 1 × 10^5^ cells per well, inoculated 96‐well platesn, and incubated for 24, 48, 72, and 96 h. After then,10 µL of CCK‐8 reagent (BL1055A, Bioshaarp) was added.Each sample was set up with 3 replicates and was incubated at 37 °C for 2 h. Cell viability was measured by the absorbance at 450 nm.

### Colony Formation Assay

Cells were seeded in 6‐well plates at a density of 1000 cells per well and cultured in medium supplemented with 10% fetal bovine serum (FBS) and 1% penicillin‐streptomycin. The cells were maintained at 37 °C in a humidified atmosphere containing 5% CO₂ for 14 days. On the final day of culture, cells were fixed with 4% paraformaldehyde for 15 min and colonies were stained with 0.1% crystal violet. The number of colonies was quantified, and images were captured under an optical microscope for further analysis.

### Edu Assay

Cell proliferation was analyzed using an EdU kit (AC11L251, Life‐ilab). Cells were seeded into 96‐well plates with a density of 1 × 10^5^ cells each well. After then, cells were incubated with 50 µm EdU buffer at 37 °C for 2 h. Subsequently, the cells were fixed with 4% paraformaldehyde for 15 min, incubated with 0.2% glycine for 10 min, and permeabilized with 0.5% Triton X‐100 for 10 min. EdU solution was introduced into the culture, followed by nuclear staining with Hoechst 33342, and the results were subsequently visualized using a fluorescence microscope.

### Cell Apoptosis Analysis

After cell transfection, 1 × 10^6^ cells were collected from each group. The cell apoptosis was detected using Annexin V‐APC/7‐AAD Apoptosis Detection Kit (E‐CK‐A218, Elabscience) according to the manufacturer's instructions. Ten microliters of Annexin V‐APC and 5 µL 7‐AAD were added to the 500 µL cell resuspension and incubated for 30 min in the dark. The percentage of apoptotic cells were detected by a CytoFLEX flow cytometer (Beckman Coulter, CA, USA).

### Transwell Assay

Cell migration was determined by a transwell assay using a transwell chamber with an 8.0‐mm pore size (Jetbiofil, China). In a cell migration assay, treated cells were placed in the upper chamber containing serum‐free RPMI 1640 medium, while the lower chamber was filled with medium supplemented with 10% fetal bovine serum (FBS) and 1% penicillin‐streptomycin. The cells were incubated for 24 h at 37 °C in a 5% CO₂ incubator. Subsequently, the cells were fixed with 4% paraformaldehyde and stained with crystal violet solution (G1059, Solarbio). Upper transwell chambers coated with Matrigel (3D200‐005, BDBIO) was used for invasion assay. The cells were then observed and photographed under an optical microscope for counting.

### Western Blot

The total protein was extracted from variously treated cells using Laemmli 2×Concentrate (S3401; Sigam). Equal amounts of protein were separated by polyacrylamide gel electrophoresis and then transferred to nitrocellulose (NC) membrane (66485, PALL). Membranes were briefly stained with a 0.1% Ponceau S solution for 5 min to verify protein transfer efficiency, followed by three washes with TBST. For blocking, membranes were incubated with 5% non‐fat milk in TBST for 1 h at room temperature. Then, these membranes were blocked with 5% non‐fat milk, probed with primary antibodies at 4 °C overnight. After three 5‐min washes with TBST, they were incubated with their corresponding secondary antibody for 2 h at room temperature. After incubation with an enhanced chemiluminescence kit (180‐506, Tanon), the protein bands were captured by a ChemiDoc Touch Imaging System (Bio‐Rad, Hercules, USA). The RIP primary antibodies are shown as follows: E‐Cadherin (ET1607‐75, HUABIO), N‐Cadherin (ET1607‐37, HUABIO), Vimentin (ET1610‐39, HUABIO), GLUT1 (ET1601‐10, HUABIO), HK2 (HA500186, HUABIO), PFKFB3 (ET1705‐66, HUABIO), PKM2 (ER1802‐70, HUABIO), LDHA (ER00702, HUABIO), Ubiquitin (ET1609‐21, HUABIO), MARCH1 (ER63906, HUABIO), MDM2 (HA601310, HUABIO), SYVN1 (HA721874, HUABIO), CBLB (HA722008, HUABIO), Pan‐Kla (HA722037, HUABIO), Histone H3 (ET1701‐64, HUABIO), K63‐linkage Ubiquitin (ET1703‐07, HUABIO), OCT4 (EM100306, HUABIO), IGF2BP2 (R389232, Zen‐bio), IGF2BP3 (200898, Zen‐bio), BMP7 (R381724, Zen‐bio), Flag (20543‐1‐AP, Proteintech), WTAP (60188‐1, Proteintech), MARCH8 (14119‐1‐AP, Proteintech), IGF2BP1 (22803‐1‐AP, Proteintech), Snail (A5243, ABclonal), β‐Actin (AC004, ABclonal), K48‐linkage Ubiquitin (A3606, ABclonal), H2BK16la (PTM‐1424RM, PTM‐BIO), H3K9la (PTM‐1419RM, PTM‐BIO), H3K14la (PTM‐1414RM, PTM‐BIO), H3K18la (PTM‐1427RM, PTM‐BIO), H4K5la (PTM‐1407RM, PTM‐BIO), H4K8la (PTM‐1415RM, PTM‐BIO), H4K12la (PTM‐1411RM, PTM‐BIO), H4K16la (PTM‐1417RM, PTM‐BIO), Histone H4 (PTM‐1015RM, PTM‐BIO).

### Fluorescence In‐Situ Hydration (FISH) and Immunofluorescence (IF)

Single FISH detection of the interaction between PDIA3P1 and miR‐152‐3p. Separate IF detection of co‐localization between HK2 and MARCH8, as well as expression of GLUT1, HK2, pan Kla, H4K8la, and BMP7. FISH and IF double staining were performed to detect the interaction of PDIA3P1 and HK2 or IGF2BP1. The Cy3‐labeled PDIA3P1 probe and FITC‐labeled miR‐152‐3p probe were synthesized and obtained from GENERALBIOL (Chuzhou, China). FISH detection Kit from GenePharma (Shanghai, China) was employed according to the manual. Cells were seeded in a confocal dish and fixed in 4% paraformaldehyde for 20 min and washed three times in PBS. Then cells were permeabilized in Triton X‐100 (P0096, Beyotime) for 5 min and blocked with 1% Bovine Serum Albumin for 30 min. Then the cells were incubated with indicated primary antibodies overnight at 4 °C. Cells were then incubated with secondary antibody at room temperature for 1 h. Simultaneously perform FISH and IF, incubate cells overnight with PDIA3P1 probe, wash, and incubate again with primary antibody. Then, after incubation with secondary antibodies. Cell nuclei were stained with DAPI (BL105A, Biosharp). Fluorescence detection of immunofluorescence Microscope (Zeiss LSM 900, Germany). The FISH probe sequences are shown as follows: PDIA3P1: 5′‐ GCAAAGACCTGAATATCGT‐3′; miR‐152‐3p: 5′‐CCAAGTTCTGTCATGCACTGA‐3′. The IF primary antibody are shown as follows: GLUT1 (ET1601‐10, HUABIO), HK2 (HA500186, HUABIO), Flag (20543‐1‐AP, Proteintech), Pan‐Kla (HA722037, HUABIO), H4K8la (PTM‐1415RM, PTM‐BIO) and BMP7 (12221‐1‐AP, Proteintech).

### RNA Immunoprecipitation (RIP)

RNA immunoprecipitation kit (P0101, Geneseed) was leveraged for RIP assay according to the manufacturer's instruction. Briefly, cells were harvested and lysed by RIP lysis buffer, then incubated with protein A/G magnet beads conjugated with the specific antibody or the IgG at 4 °C overnight. The co‐precipitated RNAs were then isolated by elution buffer, PDIA3P1 level was measured by qRT‐PCR. The RIP primary antibodies are shown as follows: AGO2 (ET1702‐39, HUABIO), Flag (20543‐1‐AP, Proteintech), WTAP (60188‐1, Proteintech), IGF2BP1 (22803‐1‐AP, Proteintech), N6‐methyladenosine (A19841, ABclonal) Mouse IgG (HA601336, HUABIO) and Rabbit IgG (HA722127, HUABIO).

### Biotin‐Labeled miRNA Pull‐Down Assay

Biotin‐labeled miRNA pull‐down assay were performed using a Biotin RNA pulldown kit (FI8702, Fitgene), following the manufacturer's instructions. Briefly, 2 × 10e7 cells were resuspended in lysis buffer and sonicated on ice, centrifuged at 12 000 g at 4 °C for 15 min, and the supernatant was collected. Then incubate Biotin‐labeled miRNA‐152‐3p and miRNA control (Fitgene) with cell lysate. Finally, Trizol (AC13984, ACMEC) was added to the magnetic beads to extract RNA, and qRT‐PCR was used to detect the abundance of PDIA3P1 in the isolated components.

### Luciferase Reporter Gene Assays

The wildtype or mutant sequences of PDIA3P1 or GLUT1 3’‐UTR were subcloned into pmirGLO vector. PDIA3P1 A‐G mutation, A‐T mutation, and A‐Del mutation were constructed by Gentlegan. ESCC cells were co‐transfected with PDIA3P1‐WT, PDIA3P1‐MUT, GLUT1 3’‐UTR‐WT, or GLUT1 3’‐UTR‐MUT as well as miR‐152‐3p mimics (HY‐R00306, MCE) or miR‐152‐3p (HY‐RI00305, MCE) inhibitor. After 48 h, cells were lysed and activities of firefly luciferase and Renilla luciferase were analyzed Dual Luciferase Reporter Gene Assay Kit (11402ES60, YEASEN) according to the manufacturer's instruction. The Firefly luciferase activity was normalized to the Renilla luciferase activity that reflects expression efficiency.

### Immunoprecipitation (IP)

The cells were collected in IP lysis buffer containing the protease inhibitor PMSF (ST505, Beyotime) and protease inhibitor cocktail (P1005, Biosharp). After 15 min of lysis, 500 µg of whole cell lysate protein was incubated by incubation with 5 µg of the Flag (20543‐1‐AP, Proteintech) antibody overnight at 4 °C, IgG as a control. The mixture was further incubated with Protein A + G Agarose (P2012, Beyotime) for 5 h, and after washing three times with cold PBS buffer, the beads were boiled in 2×SDS loading buffer. Protein expression analysis was performed by Western blot.

### RNA Pull‐Down

To prepare the DNA template for in vitro RNA synthesis, PDIA3P1 was subcloned into pcDNA3.1. PDIA3P1 were amplified by PCR (E005‐01A, Novoprotein) with primers containing F2 fragments or T7 promoter fragments, then the PCR products were recovered and transcribed with T7 High Yield RNA Transcription Kit (R7018S, Beyotime). F2‐RNA pull down kit (FI8701, Fitgene) was used for RNA pull‐down assay. Magnetic beads preparation and RNA‐binding protein extraction were performed according to the standard kit instructions. Then the retrieved proteins were then analyzed by western blot. All primer sequences are shown as follows: PDIA3P1 sense:forward: TAATACGACTCACTATAGGGAAACTAAATCAAACTTGAGTATGAAAC. Reverse: GGCGCTTTGTCAGCGCCAGTTTAAAGGGGTCTTATTTATTGTCA. Antisense: forward: GGCGCTTTGTCAGCGCCAAACTAAATCAAACTTGAGTATGAAAC. Reverse: TAATACGACTCACTATAGGGAGTTTAAAGGGGTCTTATTTATTGTCA.

### Cleavage under Targets and Tagmentation (CUT&Tag)

The CUT&Tag assay was performed using the Novo CUT&Tag High‐Sensitivity Kit (N259‐YH0, Novoprotein) for according to the manufacturer's instructions. Briefly, cells were harvested and washed in a Wash Buffer. onA magnetic bead‐bound cells were resuspended in 50 µL precooled Primary Antibody Buffer containing the appropriate primary antibody (H4K8la, PTM‐1415RM, PTM‐BIO). IgG was used as the control antibody. The primary antibody was removed, followed by incubation with the secondary antibody. Next, cells were resuspended in Tagmentation buffer and incubated at 37 °C for 1 h. Beads were added to each tube by vortexing, and quickly spun to extract the DNA. In addition, the cut DNA fragments can be ligated with P5 and P7 adaptors by Tn5 transposase, and the libraries were amplified by PCR with the P5 and P7 primers. Finally, Jingjie PTM‐Biolab (Hangzhou, China) sequenced the purified PCR products. For qPCR, purified PCR were detected according to the protocols. The primers for CUT&Tag detection are shown as follows: forward: CATTTCCCTCCCATAGCCTAACTAC. Reverse: AGCATGCCTGGTCTACTTTACTCCT.

### Xenograft Studies

About in vivo tumorigenesis model, a total of 2 × 106 stably PDIA3P1‐KD, stably PDIA3P1‐KD, and BMP7‐OE or control Eca‐109 cells were inoculated subcutaneously into fossa axillaries of five‐week‐old female nude BALB/c mice. The tumor volumes were measured every 5 days. On day 25 after injection, the mice were sacrificed and the subcutaneous tumors were excised and weighed. All tumors were paraffin‐embedded and cut into 5 µm tissue sections for subsequent analysis. Immunohistochemistry (IHC) staining was performed with an antibody specific for BMP7 (12221‐1‐AP, Proteintech) and Ki‐67 (HA721115, HUABIO). TUNEL (AC12L056, Life‐ilab) staining for detecting cell apoptosis. All animal experiments were approved by the Wannan Medical College (Approval Number: WNMC‐AWE‐2023099).

### Statistical Analysis

All experimental data are presented as the mean ± SD from three independent replicates. Statistical analysis was performed using SPSS 22.0 (IBM Corporation, Armonk, NY, USA) and GraphPad Prism 6 (La Jolla, CA, USA). Student's t‐test was used to compare the data differences between two groups. One‐way ANOVA followed by post hoc test was used to compare the data differences between three groups. *P* value < 0.05 was considered statistically significant. *P < 0.05, **P < 0.01 and ***P < 0.001.

### Ethics Approval and Consent to Participate

All experiments involving patients were approved by the Institutional Ethics Committee of The First Affiliated Hospital of Wannan Medical College (NO. 2024–66) and complied with the Declaration of Helsinki. Written informed consent was obtained from each subject. All animal experiments were approved by the Wannan Medical College (Approval Number: WNMC‐AWE‐2023099). All Institutional and National Guidelines for the care and use of animals were followed.

## Conflict of Interest

The authors declare no conflict of interest.

## Author Contributions

T.H. and Q.Y. contributed equally to this work. S.Z., W.M., and Z.W. conceived and designed the research. T.H., Q.Y., J.L., X.S., and C.W. performed the experiments. D.H., X.T., and X.X. performed the statistical analyses. Z.H. and S.Y. coordinated in all experiments and performed data analysis. HT wrote the manuscript. S.Z., W.M., and Z.W. revised the paper. All authors read and approved the final manuscript.

## Supporting information



Supporting Information

Supplemental Data 1

Supplemental Data 2

## Data Availability

The data that support the findings of this study are available from the corresponding author upon reasonable request.
