# Supplementary material for: WTAP Mediated m6A Modification Stabilizes PDIA3P1 and Promotes Tumor Progression Driven by Histone Lactylation in Esophageal Squamous Cell Carcinoma
Source: Adv Sci (Weinh). 2025 Jun 5;12(33):e06529. doi: 10.1002/advs.202506529 (PMC12412560; doi:10.1002/advs.202506529)
Supplement: Supplementary file 1 — Supporting Information [file ADVS-12-e06529-s003.pdf]

ADVANCED SCIENCE
Open Access

Supporting Information

for *Adv. Sci.*, DOI 10.1002/advs.202506529

WTAP Mediated m6A Modification Stabilizes PDIA3P1 and Promotes Tumor Progression Driven by Histone Lactylation in Esophageal Squamous Cell Carcinoma

Tao Huang, Qi You, Jiawei Liu, Xuguang Shen, Dengjun Huang, Xinlu Tao, Zhijie He, Chengwei Wu, Xinran Xi, Shouqiang Yu, Feng Liu, Zhihao Wu, Wenjun Mao* and Shaojin Zhu**

Supplementary Material for

WTAP Mediated m6A Modification Stabilizes PDIA3P1 and Promotes Tumor Progression Driven by Histone Lactylation in Esophageal Squamous Cell Carcinoma

The PDF file includes:

Supplementary Figure 1-6

Supplementary Table 1-5

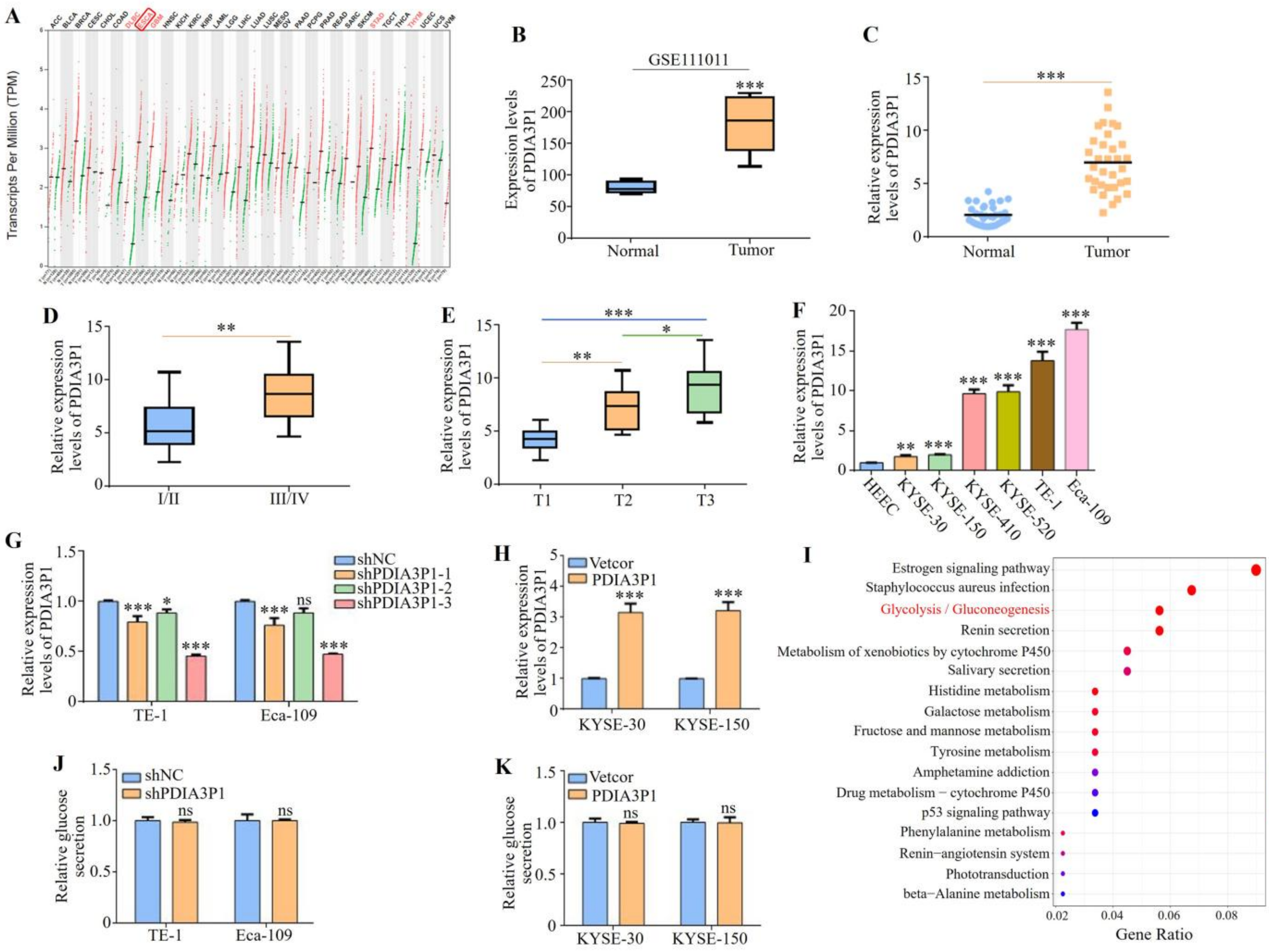

Figure S1. Supplementary figures of Figure 1

A) PDIA3P1 expression level in GEPIA database. **B)** Analysis of PDIA3P1 expression in GEO datasets (GSE111011). **C)** qRT-PCR was used to detect the relative expression of PDIA3P1 in 33 paired ESCC tissues and non-tumor specimens. **D)** Expression levels of PDIA3P1 were compared between patients in the TNM stage I/II group ($n = 18$) and the TNM III/IV group ($n = 15$). **E)** PDIA3P1 expression levels were assessed in the T1 group ($n = 10$), the T2 group ($n = 11$) and T3 group ($n = 12$). **F)** Expression levels of PDIA3P1 in normal cell line HEEC and six ESCC cell lines (KYSE-30, KYSE-150, KYSE-520, KYSE-410, TE-1 and Eca-109) were examined using qRT-PCR. **G, H)** The relative expression of PDIA3P1 in TE-1 and Eca-109 cell lines with stably silenced PDIA3P1 (G) or with stably forced-expressed PDIA3P1 (H) by qRT-PCR. **I)** KEGG analysis of the relationship between PDIA3P1 expression and related pathways in TCGA database. **J, K)** Cellular glucose secretion was measured in cells described in knocking down PDIA3P1 cells (J) or overexpressing PDIA3P1 cells (K) using a glucose assay kit. These data represent the mean ± S.D. of triplicates. *P < 0.05; **P < 0.01; ***P < 0.001.

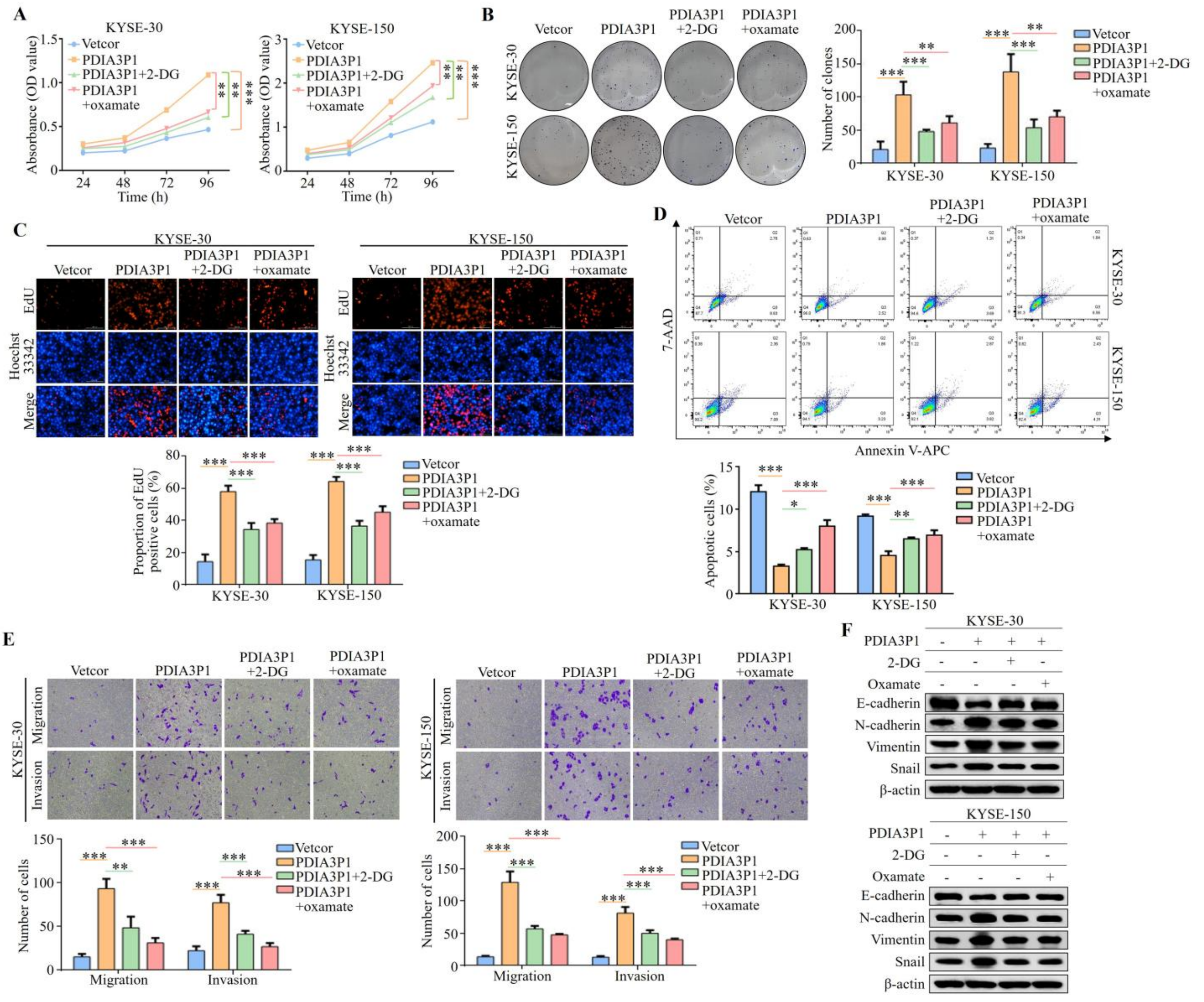

Figure S2. Supplementary figures of Figure 2

A-F) Two ESCC cell lines KYSE-30 and KYSE-150 cells stably overexpressed PDIA3P1 were treated with glycolysis inhibitors Oxamate (20mM) or 2-DG (10mM) for 24 h. (**A-C)** Using CCK-8 assay **(A)**, colony formation assay **(B)** and EdU assays **(C)** detection of cell proliferation ability. EdU scale bar: 100 µm. (**D)** Cell apoptosis detected by flow cytometry. **(E)** Transwell assays were examined cell migration and invasion. Transwell scale bar: 10 µm. (**F)** Western blot shows expression levels of E-Cadherin, N-Cadherin, Vimentin and Snail. These data represent the mean ± S.D. of triplicates. $*P < 0.05$; $**P < 0.01$; $***P < 0.001$.

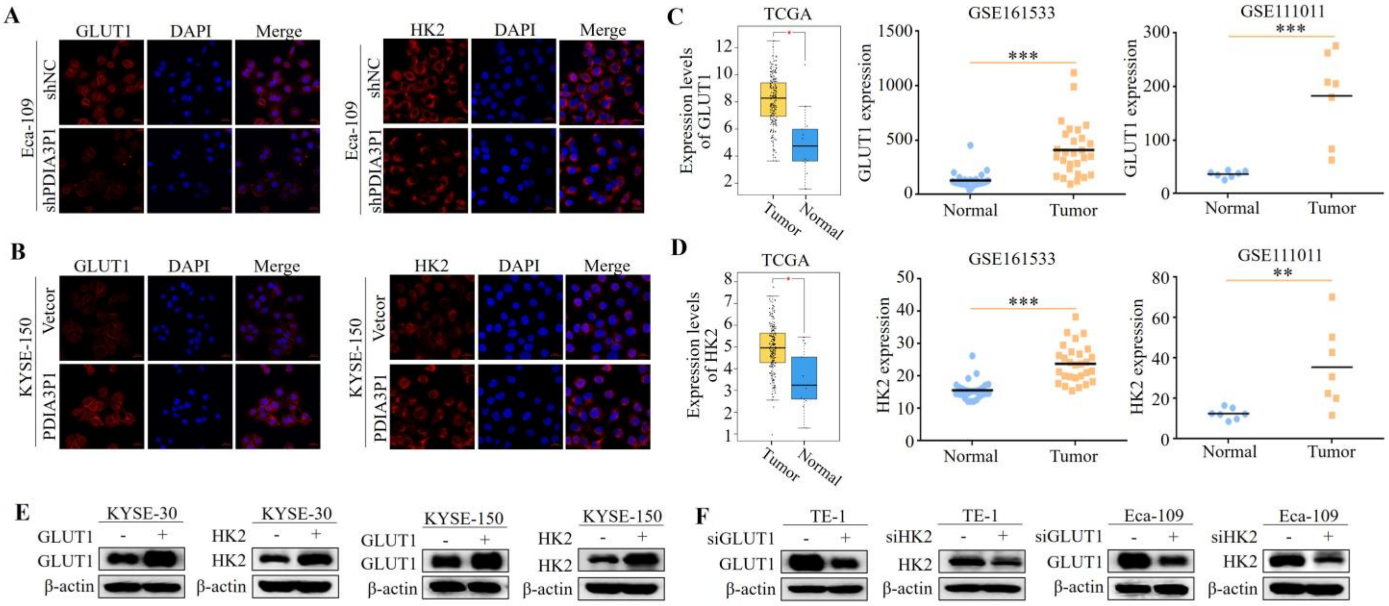

Figure S3. Supplementary figures of Figure 3

A) Representative images of immunofluorescence staining revealing the effect of PDIA3P1 knockdown on the expression of GLUT1 and HK2 in Eca-109. Scale bar: 20 μm. **B)** Representative images of immunofluorescence staining revealing the effect of PDIA3P1 overexpressed on the expression of GLUT1 and HK2 in KYSE-150. Scale bar: 20 μm. **C, D)** GLUT1 (C) and HK2 (D) expression level in GEPIA database and GEO database. **E)** KYSE-30 and KYSE-150 cell lines were transfected with cDNA of GLUT1 and HK2, the expression of GLUT1 and HK2 were analyzed by western blotting. **F)** TE-1 and Eca-109 cell lines were transfected with siGLUT1 and siHK2, the expression of GLUT1 and HK2 were analyzed by western blotting.

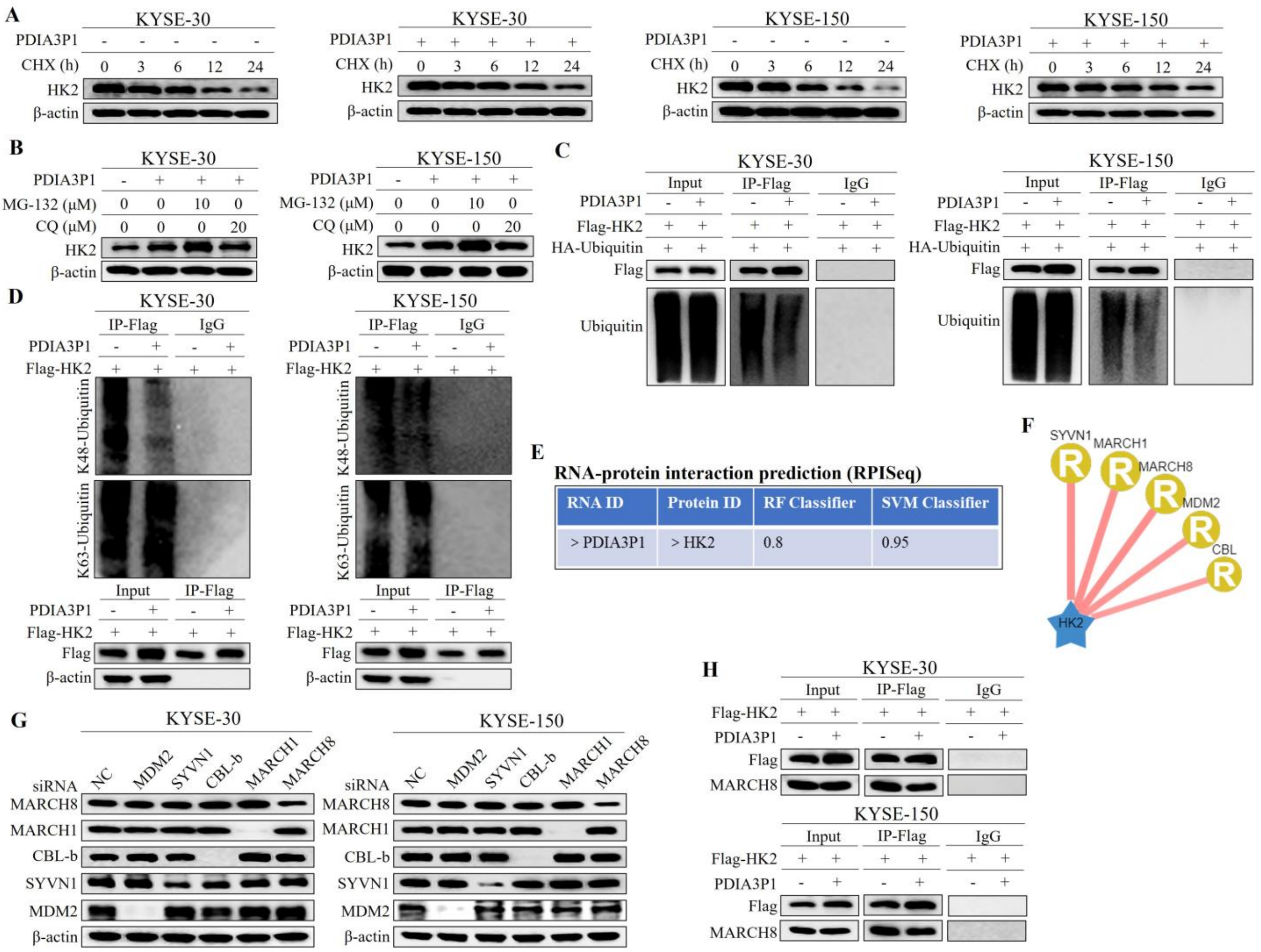

Figure S4. Supplementary figures of Figure 5

A) CHX assay for the half-life of HK2 protein in PDIA3P1-overexpression ESCC cells by Western blot. **B)** Western blot analysis expression of HK2 after treatment with MG132 (10 μM) or CQ (20 μM) in PDIA3P1-OE cells. **C)** The ubiquitination level of HK2 was detected by Western blot in cells that stabilized over-expressed PDIA3P1. IgG was used as a negative control. **D)** PDIA3P1-OE cells were co-immunoprecipitated with Flag antibody, and the expression of K48-linked ubiquitin and K63-linked ubiquitin in the immune complex was detected. **E)** Online prediction of PDIA3P1 interacts with HK2 by RPISeq. **F)** UbiBrowser was used to analyze the E3 ligase that interacts with HK2. **G)** siRNA of SYVN1, MARCH1, MARCH8, MDM2, and CBL-b were transfected into KYSE-30 and KYSE-150 cells, respectively, and the knockdown efficiency was detected by Western blot assay. **H)** Transfection of Flag-HK2 plasmid into cells, the effect of PDIA3P1 overexpression on the interaction between HK2 and MARCH8 was determined by co-IP assay in ESCC cells.

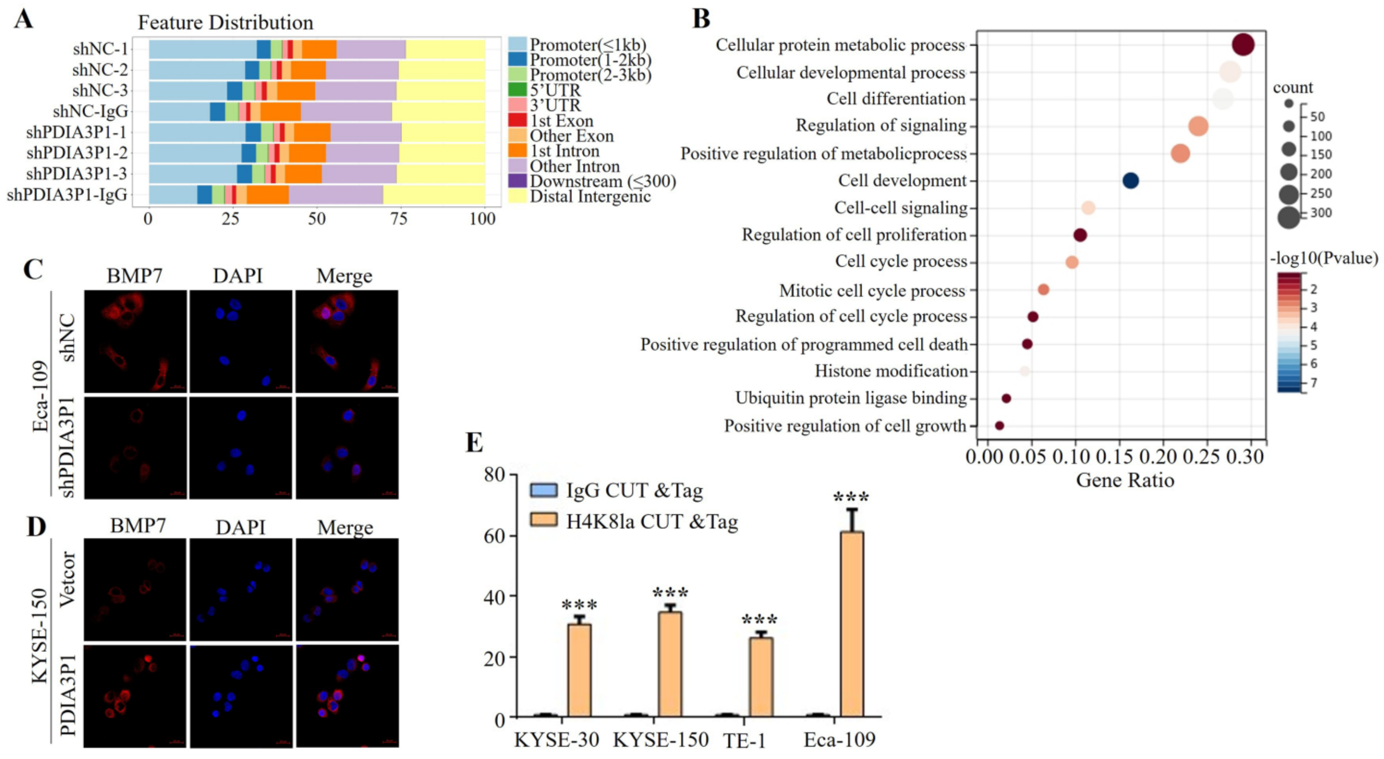

Figure S5. Supplementary figures of Figure 7

A) Bar chart of Peak functional area annotation distribution for all samples. **B)** GO analysis of the decreased H4K8la binding peaks at candidate target genes. **C, D)** Representative images of IF staining revealing the effect of PDIA3P1 knockdown in Eca-109 (C) or PDIA3P1 overexpressed in KYSE-150 (D) on the expression of BMP7. **E)** KYSE-30, KYSE-150, TE-1 and Eca-109 cells were subjected to CUT&Tag assays by using anti-H4K8la antibodies or control IgG. These data represent the mean ± S.D. of triplicates. ***$P < 0.001$.

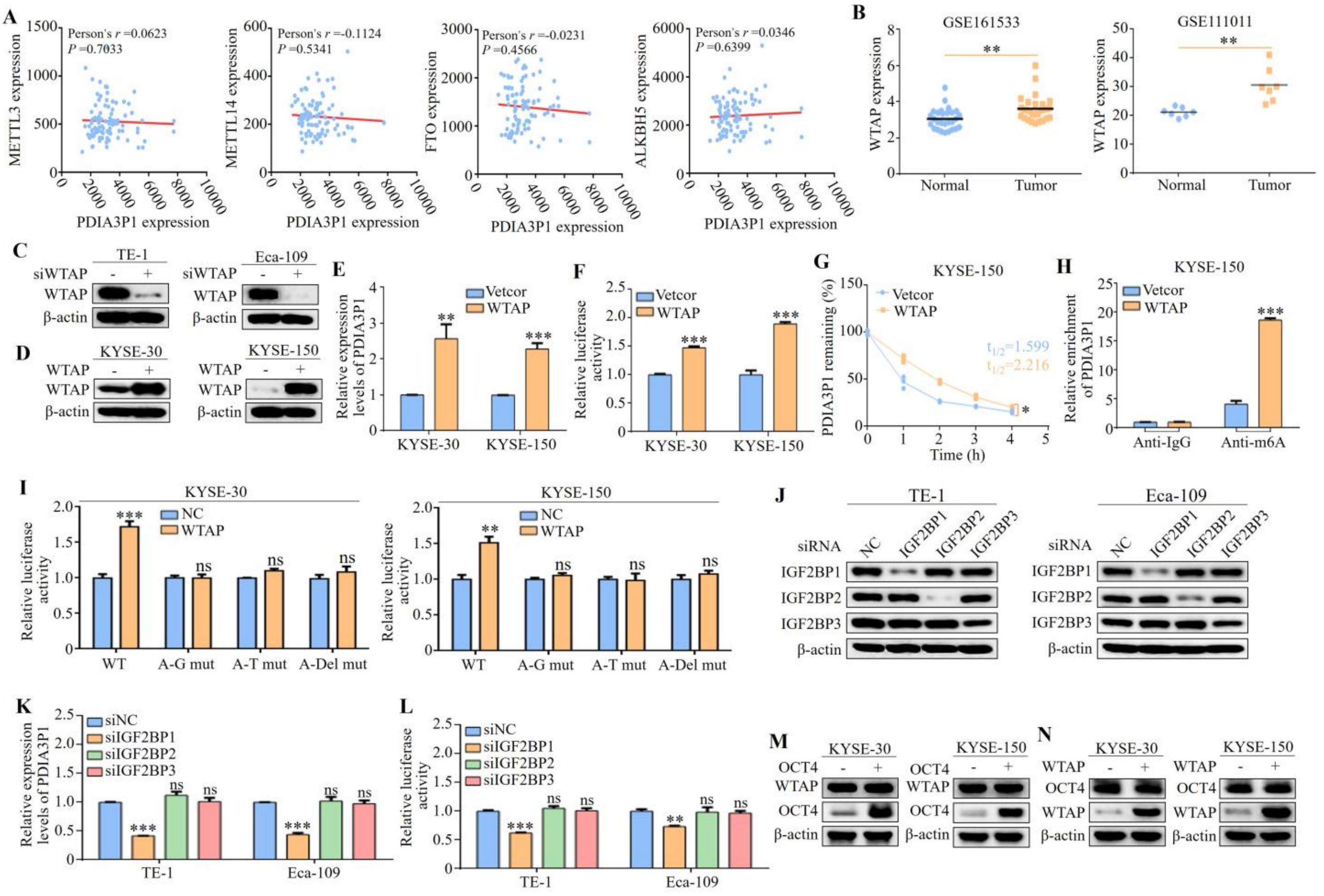

Figure S6. Supplementary figures of Figure 9

A) Correlation analysis between METTL3, METTL14, FTO and ALKBH5 with PDIA3P1 expression. B) The expression of WTAP in GEO database (GSE161537 and GSE111011). **C)** TE-1 and Eca-109 cell lines were transfected with siWTAP, the expression of WTAP was analyzed by Western blotting. **D)** KYSE-30 and KYSE-150 cell lines were transfected with cDNA of WTAP, the expression of WTAP was analyzed by Western blotting. **E)** qRT-PCR analysis of PDIA3P1 expression in KYSE-30 and KYSE-150 cells with WTAP overexpression. **F)** Relative luciferase activity in KYSE-30 and KYSE-150 cells co-transfected with luciferase reporter pmirGLO-PDIA3P1 and WTAP cDNA. **G)** WTAP overexpressing cells treated with actinomycin D (10 µg/mL) for the various time points; the level of PDIA3P1 was examined by qRT-PCR. **H)** The m6A modification level of PDIA3P1 was examined in WTAP overexpressing cells by MeRIP-qPCR. **I)** Relative luciferase activity of the wild-type and its mutants pmirGLO-PDIA3P1 reporter vectors during WTAP overexpressing in ESCC cells. **J)** siRNA of IGF2BP1, IGF2BP2 and IGF2BP3 were transfected into TE-1 and Eca-109 cells, respectively, and the knockdown efficiency was detected by Western blot assay. **K)** qRT-PCR analysis of PDIA3P1 expression in TE-1 and Eca-109 cells with or without IGF2BP1, IGF2BP2 or IGF2BP3 silencing. **L)** Relative luciferase activity in TE-1 and Eca-109 cells co-transfected with luciferase reporter pmirGLO-PDIA3P1 and IGF2BP1, IGF2BP2 or IGF2BP3 siRNA. **M)** Western blotting analysis of WTAP

expressions in KYSE-30 and KYSE-150 cells with OCT4 overexpression. **N)** Western blotting analysis of OCT4 expressions in ESCC cells with WTAP overexpression. These data represent the mean ± S.D. of triplicates. ns: no significance; **P < 0.01; ***P < 0.001.

Table S1. The correlation between clinicopathological characteristics and PDIA3P1 expression level in 33 esophageal squamous cell carcinoma patients.

**Characteristics**	**No. of patients (%)**	**PDIA3P1 expression**		***P*** [a]
		**Low *n* = 17 (%)**	**High *n* = 16 (%)**	
Age				0.708
<60 years	10 (30.3)	6 (35.3)	4 (25.0)	
≥60 years	23 (69.7)	11 (64.7)	12 (75.0)	
Gender				1.000
Female	6 (18.2)	3 (17.6)	3 (18.8)	
Male	27 (81.8)	14 (82.4)	13 (81.2)	
Differentiation				0.026*
Well	11 (33.3)	9 (52.9)	2 (14.3)	
Moderate/Poor	22 (66.7)	8 (47.1)	14 (85.7)	
TNM stage				0.015*
I/II	18 (54.5)	13 (76.5)	5 (31.3)	
III/IV	15 (45.5)	4 (23.5)	11 (68.7)	
T grade				0.032*
T1-2	21 (63.6)	14 (82.4)	7 (46.2)	
T3-4	12 (36.4)	3 (17.6)	9 (53.8)	
Lymph node metastasis				0.296
No	14 (42.4)	9 (52.9)	5 (43.8)	
Yes	19 (57.6)	8 (47.1)	11 (56.2)	

[a] Chi-square test results, *P < 0.05.

Table S2. Sequences of siRNAs.

Gene	Primer (5'>3')
PDIA3P1	CAACGAUUAGAGGACACUATT
GLUT1	AUCAUCAGCAUUGAAUUCCTT
HK2	GCUUGAAGAUUAGGUACUAUCTT
MDM2	AAGGAAUAAGCCCUGCCCATT
SYVN1	CAGGCUUCAUCAAGGUUCUTT
CBL-b	UUUGCUAACGGACCAGUACUUTT
MARCH1	GGUAGUGCCUGUACCACAATT
MARCH8	GGAAGAGACUCAAGGCCUATT

WTAP	GGGCAACACAACCGAAGAUTT
IGF2BP1	CCAAAGUUCGUAUGGUUAUTT
IGF2BP2	CAUGCCGCAUGAUUCUUGATT
IGF2BP3	GCUGCUGAGAAGUCGAUUATT

Table S3. Sequences of shRNA.

Gene	Primer (5'>3')
PDIA3P1-1#	ACTAAATCAAACTTGAGTATG
PDIA3P1-2#	CATGTGCCTCCGCCGCCCAGC
PDIA3P1-3#	GTGCTAGGACTCAGGGACGAC

Table S4. Primers used for qRT-PCR.

Gene	Primer (5'>3')
PDIA3P1	Forward: GGAAAACCACTGGGGAGGAC<br>Reverse: CAGTGCAGCTAAGAAATGGCT
GLUT1	Forward: ATTGGACAGGCTCAAAGAGG<br>Reverse: AAATGAACACAGGGCAGCTT
HK2	Forward: GTGAATCGGAGAGGTCCCAC<br>Reverse: CAAGCAGATGCGAGGCAATC
β-actin	Forward: CCTTCCTGGGCATGGAGTCCT<br>Reverse: GGAGCAATGATCTTGATCTTC
miR-148a-3p	Forward: GCTCAGTGCACTACAGAA
miR-148b-3p	Forward: CGCGTCAGTGCATCACAGAA
miR-152-3p	Forward: CCCTCAGTGCATGACAGAACTTG
miR-3064-5p	Forward: GCGGCGGTCTGGCTGTTGTGGT
miR-6504-5p	Forward: CCTTCCTGGGCATGGAGTCCT
miRNA	Reverse: CGCGGACGTAATGTCGTGT

Table S5. Primers used for miRNAs reverse transcription.

Gene	Primer (5'>3')
miR-148a-3p	GTCGTATCCAGTGCAGGGTCCGAGGTATTCGCACTGGATACGACACAAAG
miR-148b-3p	GTCGTATCCAGTGCAGGGTCCGAGGTATTCGCACTGGATACGACACAAAG
miR-152-3p	GTCGTATCCAGTGCAGGGTCCGAGGTATTCGCACTGGATACGACCCAAGTT
miR-3064-5p	GTTGGCTCTGGTGCAGGGTCCGAGGTATTCGCACCAGAGCCAACTTGCAC
miR-6504-5p	GTCGTATCCAGTGCAGGGTCCGAGGTATTCGCACTGGATACGACAGACCG
